# Concise Synthesis of Pseudane IX, Its *N*-Oxide, and Novel Carboxamide Analogs with Antibacterial Activity

**DOI:** 10.3390/molecules29153676

**Published:** 2024-08-02

**Authors:** Plamen Angelov, Yordanka Mollova-Sapundzhieva, Francisco Alonso, Bogdan Goranov, Paraskev Nedialkov, Denitsa Bachvarova

**Affiliations:** 1 Department of Organic Chemistry, University of Plovdiv Paisii Hilendarski, 24 Tsar Asen Str., 4000 Plovdiv, Bulgaria; danimollova1976@gmail.com (Y.M.-S.); denica_bachvarova@abv.bg (D.B.); 2 Instituto de Síntesis Orgánica and Departamento de Química Orgánica, Facultad de Ciencias, Universidad de Alicante, Apdo. 99, 03080 Alicante, Spain; falonso@ua.es; 3 Department of Microbiology, University of Food Technologies, 26 Maritza Boulevard, 4002 Plovdiv, Bulgaria; b_goranov@uft-plovdiv.bg; 4 Department of Pharmacognosy, Faculty of Pharmacy, Medical University of Sofia, 2 Dunav Str., 1000 Sofia, Bulgaria; pnedialkov@pharmfac.mu-sofia.bg

**Keywords:** pseudane IX, 2-nonyl-4-quinolone, NQNO, tautomerism, *Pseudomonas* metabolites, antibacterial activity

## Abstract

A four-step synthesis of the natural product pseudane IX, starting from 3-oxododecanoic acid phenylamide and including only one chromatographic purification, was accomplished with an overall yield of 52%. The same synthetic sequence, but with a controlled partial reduction of a nitro group in the penultimate intermediate, led to the *N*-oxide of pseudane IX (NQNO). A shortened three-step variation of the synthesis allowed for the preparation of novel carboxamide analogs of the natural product. An agar diffusion assay against six different bacterial strains revealed significant antibacterial activity of the novel analogs against *S. aureus* at a concentration of 100 µg/mL. One of the novel compounds showed a remarkably broad spectrum of antibacterial activity, comparable to that of the positive control NQNO.

## 1. Introduction

The natural product pseudane IX, or 2-nonyl-4-quinolone, was isolated for the first time by Hays et al. from cultures of *Pseudomonas aeruginosa* [1] and its structure was determined later by Wells, who accomplished the first synthesis of this compound [2]. Since then, pseudane IX has been isolated from various *Pseudomonas* species [3,4] and, also, from plants such as *Vepris ampody* (Rutaceae) [5] and *Ruta angustifolia* [6]. An interesting spectrum of biological activities has been reported for pseudane IX, including swarming motility inhibition in *Bacillus atrophaeus* [7], activity against *Plasmodium falciparum* [4,8] and other protozoa [8], and inhibition of *Candida albicans* biofilm formation [9]. Notably, pseudane IX has shown remarkable antiviral activity against the hepatitis C virus, exceeding that of the standard drug ribavirin [6]. The *N*-oxide of pseudane IX (NQNO) is also a known bacterial metabolite with antibiotic activity [3,10,11]. The natural sources of pseudane IX do not offer convenient isolation and sufficiently large amounts of this compound. For this reason, most of the above-cited biological studies relied on synthetic pseudane IX. The only method used for this purpose until now is based on the classic Conrad–Limpach reaction, in which an enamino ester undergoes ring closure at 270 °C in refluxing diphenyl ether [2,7,8,9,11]. The overall yield of this approach is low, and the harsh conditions impose certain limitations on the preparation of functionalized analogs. The potential of pseudane IX as a lead compound in the search for novel antimicrobials has motivated us to attempt a new synthesis of this natural product, by applying our recently published method for the preparation of 2-alkyl-4-quinolones [12]. This method is operationally simpler than the classic approach, employs milder reaction conditions, and has the additional advantage of providing access to 3-carboxamide analogs.

## 2. Results and Discussion

**Scheme 1:**
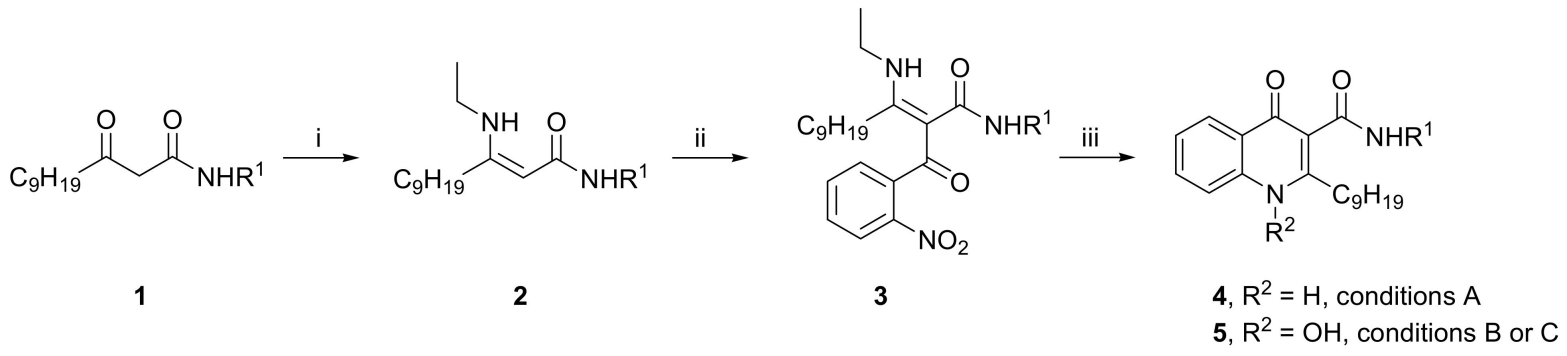
Reagents and conditions: (i) *EtNH*_2_ (70% aq, 1.05–1.15 equiv), *CH*_2_*Cl*_2_, *Na*_2_*SO*_4_, 24 h, rt; (ii) NMM (1 equiv), DMAP (0.2 equiv), 2-nitrobenzoyl chloride (1 equiv), *CH*_2_*Cl*_2_, 2 h, rt; (iii) either A: *Zn*/*HOAc*/*CH*_2_*Cl*_2_, rt, overnight, B: *HCOONH*_4_, *Pd*/*C*, *CH*_3_*OH*, rt, or C: *H*_2_ (balloon), 5 wt% *Pt*/*Al*_2_*O*_3_, *n-BuNH*_2_, DMSO, isopropanol, 24 h, rt.

In order to synthesize a few structural analogs of pseudane IX along with the targeted natural product, we first prepared three different amides of 3-oxododecanoic acid (**1a**–**c**). This was performed by acylation/deacetylation of the corresponding commercially available acetoacetamides, following our published method [13]. The β-keto amides **1** were then condensed with ethylamine, to provide enamines **2**, which were directly subjected to acylation with 2-nitrobenzoyl chloride (Scheme 1). This way, good yields of the intermediates **3** were obtained (80–85%). Next, a reduction of the nitro group in intermediates **3** with *Zn* in *CH*_2_*Cl*_2_/*HOAc* was carried out. This was accompanied by spontaneous cyclization of the reduced intermediates to the corresponding 2-nonyl-4-quinolone-3-carboxamide derivatives **4** (R^2^ = H). Compared with our previous experiments on similar substrates with shorter alkyl chains [12], the full conversion of **3** to **4** here was slightly slower and required a larger excess of the reducing agent. Alternatively, controlled partial reduction of the nitro group under *Pd*- or *Pt*-catalyzed hydrogenation conditions led to the *N*-hydroxy derivatives **5** (R^2^ = OH). Compounds **5a**,**b** were obtained by *Pd*-catalyzed transfer hydrogenation of **3a**,**b** with ammonium formate, while compound **3c** was hydrogenated with *H*_2_ over DMSO-inhibited *Pt*/*Al*_2_*O*_3_ [14] to give **5c** without concomitant reduction at the *C–Cl* bond. This way, a set of six new analogs of the natural product were prepared (Scheme 1, Table 1).

**Scheme 2:**
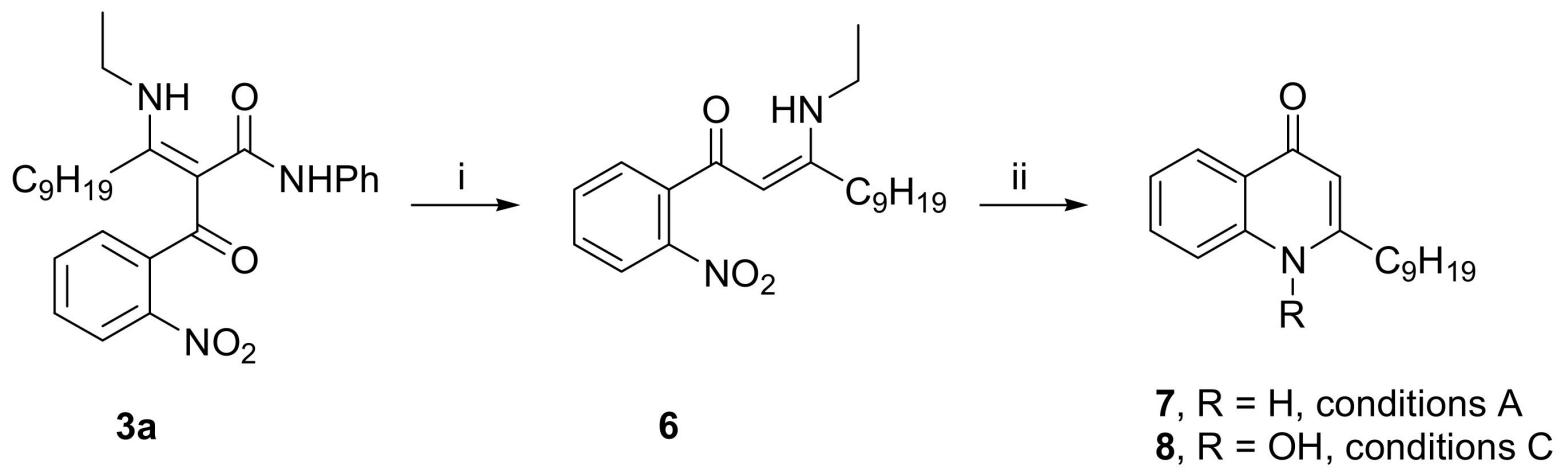
Synthesis of pseudane IX (**7**) and its *N*-oxide (**8**) from intermediate **3a**. Reagents and conditions: (i) *H*_3_*PO*_4_, 60 °C, 2 h; (ii) either A: *Zn*/*HOAc*/*CH*_2_*Cl*_2_, rt, overnight, or C: *H*_2_ (balloon), 5 wt% *Pt*/*Al*_2_*O*_3_, *n-BuNH*_2_, DMSO, isopropanol, 24 h, rt.

The synthesis of pseudane IX required one additional decarbamoylation step after the acylation stage (Scheme 2). In theory, any of the nitrobenzoylated intermediates **3** should be susceptible to such decarbamoylation and would give the same β-enamino ketone **6** upon heating in neat *H*_3_*PO*_4_ [15]. However, we only used intermediate **3a** for this purpose as it offered the best atom economy and the lowest overall price of the synthesis. This reaction was carried out by stirring compound **3a** in neat *H*_3_*PO*_4_ for 2 h at 60 °C. It should be noted that the time needed for the completion of the reaction was longer than previously reported by us for similar substrates with shorter alkyl chains [12]. The β-enamino ketone **6** was obtained in an 86% yield and its ^1^*H* NMR spectrum in DMSO-d_6_ indicated a mixture of *Z*/*E* isomers in a ratio of 85/15. In the last step, the β-enamino ketone **6** successfully underwent a reduction of the nitro group with *Zn* in *CH*_2_*Cl*_2_/*HOAc* and the ensuing spontaneous cyclization completed the synthesis of pseudane IX in 72% yield (**7**, Scheme 2). To stop the reduction at the *N*-oxide level, we carried out atmospheric pressure hydrogenation of the β-enamino ketone **6** with *H*_2_ over DMSO-inhibited *Pt*/*Al*_2_*O*_3_ [14]. Under these conditions, the *N*-oxide of pseudane IX (**8**) was cleanly obtained in 70% yield.

**Scheme 3:**
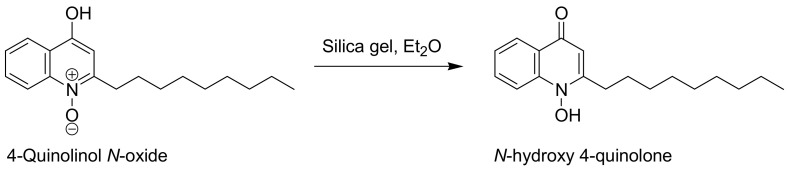
Tautomeric change of compound **8** (NQNO) during chromatography on silica gel.

Depending on the method of isolation, compound **8** was obtained in two distinctly different forms. When the crude product was only triturated with diethyl ether, it solidified as a white powder with good solubility in DMSO and a mp of 103–104 °C. The NMR spectrum of this material was taken in DMSO-d_6_ at 25 °C and was indicative of a 4-quinolinol-*N*-oxide tautomeric form (Scheme 3), with the C3-H signal appearing at 7.08 ppm. On the other hand, when compound **8** passed through a silica gel column with diethyl ether as the eluent, it crystallized as colorless needles with a mp of 146–147 °C and a very poor DMSO solubility at 25 °C. Because of the poor solubility, the NMR spectrum in DMSO-d_6_ had to be run at 70 °C, and this time it clearly indicated an *N*-hydroxy-4-quinolone tautomeric form, with the C3-H signal appearing at 5.97 ppm (See Supplementary Information). A similar change was not observed in compound **7**, which was registered only as the 4-quinolone tautomer, regardless of the isolation method.

The antibacterial activity of the novel analogs **4** and **5** was tested at a concentration of 100 µg/mL against a set of six bacterial strains, using the agar diffusion method. The known natural compounds pseudane IX (**7**) and its *N*-oxide (**8**) were also included in the assay for comparison. The *N*-oxide **8** is known for its antibiotic activity [10,11] and served as the positive control. The observed inhibition zones (Table 2) indicated higher activity of the *N*-hydroxy derivatives **5** compared with that of their reduced counterparts **4**, with *S. aureus* ATCC 25923 being the most susceptible among the studied bacteria. Notably, there was a sharp contrast in the susceptibility of the other assayed *S. aureus* strain (ATCC 6538), which was moderately inhibited by only one of the novel compounds (**5c**). Compound **5c** was also the one with the broadest activity spectrum, inhibiting all of the studied bacteria and approaching the activity of the natural antibiotic **8**. Against the resilient *S. aureus* strain (ATCC 6538), **5c** even outperformed **8** with a slightly larger inhibition zone.

In conclusion, we have accomplished a convenient synthesis of two natural products with interesting biological profiles—pseudane IX and its *N*-oxide. The overall yield over four steps, starting from the phenylamide of 3-oxododecanoic acid and involving only one chromatographic purification, is 52% and 50% respectively. We have also demonstrated that the synthetic method used for this purpose allows for easy access to novel carboxamide analogs of these compounds. A broad antibacterial spectrum was observed in one of the newly obtained analogs, providing a lead for further structural optimization.

## 3. Materials and Methods

All reagents and solvents were purchased from Sigma-Aldrich, Darmstadt, Germany, and were used as supplied. Petrol refers to the 40–60 °C fraction, *HOAc* refers to acetic acid, and NMM refers to *N*-methylmorpholine. NMR spectra were run on a Bruker NEO 400 (400/100 MHz ^1^H/^13^C) spectrometer. Chemical shifts (δ, ppm) are downfield from TMS. TLC was performed on aluminum-backed Silica gel 60 sheets (Merck) with KMnO_4_ staining. Melting point measurements were performed in capillary tubes on KRÜSS M5000 automatic mp meter and were not corrected. Mass spectral measurements were performed on a Thermo Fisher Scientific Q Exactive Plus high-resolution mass spectrometer with a heated electrospray ionization source (HESI-II). The agar diffusion antibacterial assay was carried out in LBG agar, in 6 mm wells, with 60 µL loading of 100 µg/mL solutions of the tested compounds in DMSO, according to a published protocol [16].

The starting amides of 3-oxododecanoic acid (**1a**–**c**) were prepared according to a previously published method [13], then the conversion of **1** to α-C-acylated β-enamino amides **3** was achieved according to another published general procedure [12].

*3-Ethylamino-2-(2-nitrobenzoyl)-dodec-2-enoic acid phenylamide (***3a***)*: yellowish oil; ^1^H-NMR (400 MHz, DMSO-d_6_, δ ppm, *J* Hz): 0.83 (t, *J* = 7.0, 3H, CH_3_), 1.12–1.24 (m, 12H, 6 × CH_2_), 1.27 (t, *J* = 7.2, 3H, NHCH_2_CH_3_), 1.60 (m, 2H, βCH_2_), 2.45 (m, 2H, αCH_2_), 3.50 (m, 2H, NHCH_2_CH_3_), 6.94 (m, 1H, ArH), 7.15 (m, 2H, ArH), 7.27 (m, 2H, ArH), 7.50 (m, 2H, ArH), 7.65 (td, *J* = 7.5, *J* = 1.2, 1H, ArH), 7.96 (dd, *J* = 8.2, *J* = 1.0, 1H, ArH), 9.70 (br s, 1H, CONH), 11.62 (t, *J* = 5.6, 1H, NHCH_2_CH_3_); ^13^C-NMR (100 MHz, DMSO-d_6_, δ ppm): 14.38, 15.77, 22.53, 27.94, 28.81, 29.05, 29.16, 29.51, 29.73, 31.65, 38.04, 107.58, 119.62, 123.62, 124.27, 128.78, 128.83, 129.84, 133.90, 137.70, 139.47, 146.46, 167.14, 169.33, 186.00; HRMS *m*/*z* (ES+): calcd. for C_27_H_36_N_3_O_4_^+^ [M+H]^+^ 466.2700, found 466.2704.
*3-Ethylamino-2-(2-nitrobenzoyl)-dodec-2-enoic acid 4-methoxyphenylamide (***3b***)*: yellowish oil; ^1^H-NMR (400 MHz, DMSO-d_6_, δ ppm, *J* Hz): 0.82 (t, *J* = 7.0, 3H, CH_3_), 1.12–1.24 (m, 12H, 6 × CH_2_), 1.26 (t, *J* = 7.2, 3H, NHCH_2_CH_3_), 1.60 (m, 2H, βCH_2_), 2.44 (m, 2H, αCH_2_), 3.49 (m, 2H, NHCH_2_CH_3_), 3.66 (s, 3H, OCH_3_), 6.73 (m, 2H, ArH), 7.15 (m, 2H, ArH), 7.50 (m, 2H, ArH), 7.66 (td, *J* = 7.5, *J* = 1.2, 1H, ArH), 7.96 (dd, *J* = 8.2, *J* = 0.9, ArH), 9.52 (br s, 1H, CONH), 11.60 (t, *J* = 5.5, 1H, NHCH_2_CH_3_); ^13^C-NMR (100 MHz, DMSO-d_6_, δ ppm): 14.37, 15.78, 22.54, 27.95, 28.83, 29.08, 29.18, 29.54, 29.69, 31.67, 38.01, 55.52, 113.98, 121.24, 124.25, 128.82, 129.81, 132.60, 133.85, 137.72, 146.46, 155.72, 166.70, 169.20, 185.94; HRMS *m*/*z* (ES+): calcd. for C_28_H_38_N_3_O_5_^+^ [M+H]^+^ 496.2806, found 496.2801.
*3-Ethylamino-2-(2-nitrobenzoyl)-dodec-2-enoic acid 4-chlorophenylamide (***3c***)*: yellowish oil; ^1^H-NMR (400 MHz, DMSO-d_6_, δ ppm, *J* Hz): 0.82 (t, *J* = 7.1, 3H, CH_3_), 1.10–1.22 (m, 12H, 6 × CH_2_), 1.27 (t, *J* = 7.2, 3H, NHCH_2_CH_3_), 1.59 (m, 2H, βCH_2_), 2.44 (m, 2H, αCH_2_), 3.50 (m, 2H, NHCH_2_CH_3_), 7.21 (m, 2H, ArH), 7.32 (m, 2H, ArH), 7.49 (m, 2H, ArH), 7.65 (m, 1H, ArH), 7.96 (m, 1H, ArH), 9.87 (br s, 1H, CONH), 11.62 (t, *J* = 5.6, 1H, NHCH_2_CH_3_); ^13^C-NMR (100 MHz, DMSO-d_6_, δ ppm): 14.37, 15.77, 22.54, 27.86, 28.74, 29.07, 29.14, 29.43, 29.64, 31.65, 38.07, 120.91, 124.28, 127.19, 128.78, 129.88, 133.93, 137.57, 138.46, 146.40, 167.28, 186.02; HRMS *m*/*z* (ES+): calcd. for C_27_H_35_ClN_3_O_4_^+^ [M+H]^+^ 500.2311, found 500.2318.
*Synthesis of 3-Ethylamino-1-(2-nitrophenyl)-dodec-2-en-1-one (***6***):* Intermediate **3a** (466 mg, 1 mmol) was mixed with anhydrous *H*_3_*PO*_4_ (5–6 g) in a glass vial. The mixture was stirred intensely for two hours at 60 °C, then the vial was cooled to r.t. with tap water, and the contents were rinsed and poured into a separatory funnel with 50–70 mL of water. The product was extracted in *CH*_2_*Cl*_2_ (2 × 40 mL), the combined organic layers were dried (*Na*_2_*SO*_4_), and the solvent was removed under reduced pressure. The crude product obtained this way was sufficiently clean to be used directly in the next stage, without further purification. An analytically pure sample can be obtained by column chromatography on silica gel with *Et*_2_*O*:Petrol (1:1) as the eluent, increasing polarity to *Et*_2_*O*:Petrol (2:1). Yield: 298 mg yellowish oil (86%). The ^1^H NMR spectra of **6** in DMSO-*d*_6_ indicate a *Z*/*E* isomeric mixture in a ratio of 85:15. Only NMR signals corresponding to the major *Z* isomer are listed below. ^1^H-NMR (400 MHz, DMSO-d_6_, δ ppm, *J* Hz): 0.85 (t, *J* = 7.0, 3H, CH_3_), 1.19 (t, *J* = 7.2, 3H, NHCH_2_CH_3_), 1.23–1.39 (m, 12H, 6 × CH_2_), 1.53 (m, 2H, βCH_2_), 2.34 (m, 2H, αCH_2_), 3.39 (m, 2H, NHCH_2_CH_3_), 5.35 (s, 1H, CH), 7.57–7.70 (m, 3H, ArH), 7.81 (m, 1H, ArH), 10.94 (t, *J* = 5.6, 1H, NHCH_2_CH_3_); ^13^C-NMR (100 MHz, DMSO-d_6_, δ ppm): 14.39, 15.72, 22.56, 27.78, 29.12, 29.17, 29.24, 29.35, 31.74, 37.61, 92.26, 124.12, 129.11, 130.65, 132.79, 136.94, 148.93, 170.38, 184.65; HRMS *m*/*z* (ES+): calcd. for C_20_H_31_N_2_O_3_^+^ [M+H]^+^ 347.2329, found 347.2331.
*Synthesis of compounds* **4***,* **5**, **7**, *and* **8** *by reductive cyclization of intermediates* **3** *and* **6**. *General procedure A (preparation of products* **4a**–**c** *and* **7***)*: *Zn* powder (2 g, prewashed with 1% *HCl*, water, and acetone) was added to the corresponding nitro-intermediate **3** or **6** (1 mmol), dissolved in a mixture of *CH*_2_*Cl*_2_ (30 mL) and acetic acid (4 mL). The heterogeneous mixture was magnetically stirred for 24 h at r.t., and then the solids were filtered off with suction and rinsed thoroughly with *CH*_2_*Cl*_2_. The dichloromethane filtrate was transferred to a separatory funnel and was extracted with water (50 mL) and, then, with a saturated aqueous solution of *NaHCO*_3_ (25 mL). The organic phase was dried with anhydrous sodium sulfate, the drying agent was filtered off, and the solvent was removed under reduced pressure. The products crystallized upon trituration with *Et*_2_*O*. Where necessary, further purification can be performed by column chromatography on silica gel with *Et*_2_*O* as the eluent, increasing polarity to *Et*_2_*O*:*CH*_3_*OH* 20:1.
*General procedure B (preparation of products* **5a** *and* **5b***)*: To the corresponding nitro-intermediate **3** (100 mg) in *CH*_3_*OH* (10–15 mL), *HCOONH*_4_ (300 mg) and Pd on charcoal (10 mg, 10 w% Pd) were added. The mixture was magnetically stirred for 90 min. at r.t., and the catalyst was removed by vacuum filtration through a pad of celite on a sintered glass funnel. The celite was rinsed thoroughly with methanol, and the solvent was removed from the filtrate under reduced pressure. Then, water (50 mL) was added to the solid residue and the product was extracted in *CH*_2_*Cl*_2_ (3 × 20 mL). The combined organic layers were dried with anhydrous sodium sulfate, the drying agent was filtered off, and the solvent was removed under reduced pressure. The products crystallized upon trituration with *Et*_2_*O*. Where necessary, further purification can be performed by column chromatography on silica gel with *Et*_2_*O* as the eluent, increasing polarity to *Et*_2_*O*:*CH*_3_*OH* 20:1.
*General procedure C (preparation of products* **5c** *and* **8***)*: The corresponding nitro-intermediate **3** or **6** (0.5 mmol) was dissolved in isopropanol (IPA) (2 mL). Then, DMSO (0.013 g, 0.012 mL), *n*-butylamine (0.037 g, 0.050 mL), and 5 wt% *Pt*/*Al*_2_*O*_3_ (0.010 g) were added to the solution. The air in the reaction vessel was evacuated and replaced with *H*_2_ with the help of a three-way stopper. The *H*_2_ atmosphere was kept with a balloon for the next 24 h, while the reaction mixture was magnetically stirred at 25 °C. Then, the catalyst was filtered off through a pad of celite on a sintered glass funnel, with thorough rinsing with IPA. The IPA was removed from the filtrate on a rotary evaporator under reduced pressure. To remove any residual *n*-butylamine and DMSO, dilute aqueous *HCl* was added to the residue and the product was extracted in *CH*_2_*Cl*_2_ (2 × 30 mL). The combined organic layers were dried with anhydrous sodium sulfate, the solvent was removed under reduced pressure, and the oily residue solidified upon trituration with diethyl ether, providing practically clean products. Analytically pure samples were obtained by column chromatography on a short silica gel plug, using *Et*_2_*O* as the eluent.
*2-Nonyl-4-oxo-1*,*4-dihydroquinoline-3-carboxylic acid phenylamide (***4a***)*: white solid, mp 192–193 °C; ^1^H-NMR (400 MHz, DMSO-d_6_, δ ppm, *J* Hz): 0.83 (t, *J* = 6.9, 3H, CH_3_), 1.17–1.33 (m, 10H, 5 × CH_2_), 1.39 (m, 2H, γCH_2_), 1.72 (m, 2H, βCH_2_), 3.16 (m, 2H, αCH_2_), 7.06 (m, 1H, ArH), 7.33 (m, 2H, ArH), 7.43 (m, 1H, ArH), 7.64–7.76 (m, 4H, ArH), 8.23 (dd, *J* = 8.2, *J* = 1.1, 1H, ArH), 12.17 (br s, 1H, NH), 12.20 (s, 1H, NH); ^13^C-NMR (100 MHz, DMSO-d_6_, δ ppm): 14.40, 22.56, 29.14, 29.31, 29.52, 29.88, 31.74, 33.59, 112.38, 118.61, 120.06, 123.49, 124.84, 125.15, 125.88, 129.22, 133.08, 138.85, 139.81, 158.87, 164.58, 176.70; HRMS *m*/*z* (ES+): calcd. for C_25_H_31_N_2_O_2_^+^ [M+H]^+^ 391.2380, found 391.2387.
*2-Nonyl-4-oxo-1*,*4-dihydroquinoline-3-carboxylic acid 4-methoxyphenylamide (***4b***)*: white solid, mp 150–151 °C; ^1^H-NMR (400 MHz, DMSO-d_6_, δ ppm, *J* Hz): 0.82 (t, *J* = 6.9, 3H, CH_3_), 1.17–1.33 (m, 10H, 5 × CH_2_), 1.38 (m, 2H, γCH_2_), 1.72 (m, 2H, βCH_2_), 3.15 (m, 2H, αCH_2_), 3.74 (s, 3H, OCH_3_), 6.90 (m, 2H, ArH), 7.42 (m, 1H, ArH), 7.63 (m, 3H, ArH), 7.73 (m, 1H, ArH), 8.21 (dd, *J* = 8.1, *J* = 1.1, 1H, ArH), 12.03 (s, 1H, NH), 12.12 (br s, 1H, NH); ^13^C-NMR (100 MHz, DMSO-d_6_, δ ppm): 14.40, 22.56, 29.15, 29.17, 29.33, 29.52, 29.91, 31.75, 33.56, 55.61, 112.54, 114.34, 118.60, 121.47, 124.75, 125.14, 125.85, 132.99, 133.03, 138.88, 155.55, 158.63, 164.16, 176.62; HRMS *m*/*z* (ES+): calcd. for C_26_H_33_N_2_O_3_^+^ [M+H]^+^ 421.2486, found 421.2481.
*2-Nonyl-4-oxo-1*,*4-dihydroquinoline-3-carboxylic acid 4-chlorophenylamide (***4c***)*: white solid, mp 172–173 °C; ^1^H-NMR (400 MHz, DMSO-d_6_, δ ppm, *J* Hz): 0.83 (t, *J* = 6.9, 3H, CH_3_), 1.17–1.32 (m, 10H, 5 × CH_2_), 1.38 (m, 2H, γCH_2_), 1.71 (m, 2H, βCH_2_), 3.13 (m, 2H, αCH_2_), 7.37 (m, 2H, ArH), 7.43 (m, 1H, ArH), 7.65 (m, 1H, ArH), 7.74 (m, 3H, ArH), 8.21 (dd, *J* = 8.2, *J* = 1.1, 1H, ArH), 12.19 (br s, 1H, NH), 12.30 (s, 1H, NH); ^13^C-NMR (100 MHz, DMSO-d_6_, δ ppm): 14.39, 22.56, 29.14, 29.32, 29.49, 29.84, 31.74, 33.54, 112.24, 118.65, 121.54, 124.89, 125.11, 125.84, 126.96, 129.09, 133.12, 138.74, 138.87, 158.89, 164.73, 176.64; HRMS *m*/*z* (ES+): calcd. for C_25_H_30_ClN_2_O_2_^+^ [M+H]^+^ 425.1990, found 425.1989.
*1-Hydroxy-2-nonyl-4-oxo-1*,*4-dihydroquinoline-3-carboxylic acid phenylamide (***5a***)*: white solid, mp 162–163 °C; ^1^H-NMR (400 MHz, DMSO-d_6_, δ ppm, *J* Hz): 0.84 (t, *J* = 7.0, 3H, CH_3_), 1.17–1.32 (m, 10H, 5 × CH_2_), 1.39 (m, 2H, γCH_2_), 1.76 (m, 2H, βCH_2_), 3.12 (m, 2H, αCH_2_), 7.07 (m, 1H, ArH), 7.33 (m, 2H, ArH), 7.49 (m, 1H, ArH), 7.71 (m, 2H, ArH), 7.83 (m, 1H, ArH), 7.93 (m, 1H, ArH), 8.25 (dd, *J* = 8.1, *J* = 1.2, 1H, ArH), 11.30 (s, 1H, NH), 12.10 (br s, 1H, OH); ^13^C-NMR (100 MHz, DMSO-d_6_, δ ppm): 14.41, 22.56, 28.44, 29.00, 29.15, 29.25, 29.58, 31.73, 115.05, 115.66, 119.90, 123.61, 124.96, 125.52, 125.97, 129.16, 133.33, 139.86, 139.95, 155.74, 164.60, 173.62; HRMS *m*/*z* (ES+): calcd. for C_25_H_31_N_2_O_3_^+^ [M+H]^+^ 407.2329, found 407.2324.
*1-Hydroxy-2-nonyl-4-oxo-1*,*4-dihydroquinoline-3-carboxylic acid 4-methoxyphenylamide (***5b***)*: white solid, mp 163–164 °C; ^1^H-NMR (400 MHz, DMSO-d_6_, δ ppm, *J* Hz): 0.84 (t, *J* = 6.9, 3H, CH_3_), 1.15–1.32 (m, 10H, 5 × CH_2_), 1.38 (m, 2H, γCH_2_), 1.75 (m, 2H, βCH_2_), 3.11 (m, 2H, αCH_2_), 3.74 (s, 3H, OCH_3_), 6.91 (m, 2H, ArH), 7.47 (m, 1H, ArH), 7.63 (m, 2H, ArH), 7.82 (m, 1H, ArH), 7.92 (m, 1H, ArH), 8.24 (m, 1H, ArH), 11.15 (s, 1H, NH), 12.13 (br s, 1H, OH); ^13^C-NMR (100 MHz, DMSO-d_6_, δ ppm): 14.40, 22.57, 28.44, 29.01, 29.17, 29.27, 29.59, 31.74, 55.61, 114.28, 115.16, 115.64, 121.32, 124.87, 125.51, 125.95, 133.09, 133.23, 139.95, 155.63, 164.13, 173.57; HRMS *m*/*z* (ES+): calcd. for C_26_H_33_N_2_O_4_^+^ [M+H]^+^ 437.2435, found 437.2434.
*1-Hydroxy-2-nonyl-4-oxo-1*,*4-dihydroquinoline-3-carboxylic acid 4-chlorophenylamide (***5c***)*: white solid, mp 182–183 °C; ^1^H-NMR (400 MHz, DMSO-d_6_, δ ppm, *J* Hz): 0.83 (t, *J* = 7.0, 3H, CH_3_), 1.14–1.30 (m, 10H, 5 × CH_2_), 1.38 (m, 2H, γCH_2_), 1.75 (m, 2H, βCH_2_), 3.09 (m, 2H, αCH_2_), 7.38 (m, 2H, ArH), 7.49 (m, 1H, ArH), 7.75 (m, 2H, ArH), 7.83 (m, 1H, ArH), 7.93 (m, 1H, ArH), 8.24 (dd, *J* = 8.1, *J* = 1.2, 1H, ArH), 11.40 (s, 1H, NH), 12.13 (br s, 1H, OH); ^13^C-NMR (100 MHz, DMSO-d_6_, δ ppm): 14.40, 22.57, 28.37, 28.96, 29.16, 29.24, 29.50, 31.73, 114.93, 115.70, 121.36, 125.02, 125.50, 125.94, 127.14, 129.07, 133.39, 138.80, 139.97, 155.68, 164.77, 173.53; HRMS *m*/*z* (ES+): calcd. for C_25_H_30_ClN_2_O_3_^+^ [M+H]^+^ 441.1939, found 441.1932.
*2-Nonyl-1H-quinolin-4-one (***7***)*: white solid, mp 135–136 °C (Lit. [2] mp 138–139 °C, Lit. [3] mp 134 °C); ^1^H-NMR (400 MHz, DMSO-d_6_, δ ppm, *J* Hz): 0.83 (t, *J* = 6.7, 3H, CH_3_), 1.18–1.36 (m, 12H, 6 × CH_2_), 1.66 (m, 2H, βCH_2_), 2.58 (m, 2H, αCH_2_), 5.92 (s, 1H, C3-H), 7.27 (m, 1H, ArH), 7.54 (m, 1H, ArH), 7.61 (m, 1H, ArH), 8.04 (m, 1H, ArH), 11.50 (br s, 1H, NH); ^13^C-NMR (100 MHz, DMSO-d_6_, δ ppm): 14.39, 22.55, 28.81, 28.97, 29.12, 29.19, 29.35, 31.72, 33.72, 108.09, 118.33, 123.16, 125.10, 125.22, 131.87, 140.62, 154.02, 177.33; HRMS *m*/*z* (ES+): calcd. for C_18_H_26_NO^+^ [M+H]^+^ 272.2009, found 272.2007.
*2-Nonyl-1-oxyquinolin-4-ol (***8**, *quinolinol tautomer)*: white solid, mp 103–104 °C (Lit. [10] mp 148–149 °C, Lit. [3] mp 132 °C); ^1^H-NMR (400 MHz, DMSO-d_6_, δ ppm, *J* Hz): 0.84 (t, *J* = 6.8, 3H, CH_3_), 1.17–1.43 (m, 12H, 6 × CH_2_), 1.75 (m, 2H, βCH_2_), 3.09 (m, 2H, αCH_2_), 7.08 (s, 1H, C3-H), 7.77 (m, 1H, ArH), 8.07 (m, 1H, ArH), 8.25 (m, 1H, ArH), 8.30 (m, 1H, ArH); ^13^C-NMR (100 MHz, DMSO-d_6_, δ ppm): 14.40, 22.55, 27.55, 29.13, 29.15, 29.29, 31.73, 31.75, 105.48, 117.31, 121.35, 124.25, 127.70, 134.82, 140.03, 158.60, 167.31; HRMS *m*/*z* (ES+): calcd. for C_18_H_26_NO_2_^+^ [M+H]^+^ 288.1958, found 288.1963.
*1-Hydroxy-2-nonyl-(1H)-quinolin-4-one (***8**, *quinolone tautomer)*: white solid, mp 146–147 °C (Lit. [10] mp 148–149 °C, Lit. [3] mp 132 °C); ^1^H-NMR (400 MHz, DMSO-d_6_, 70 °C, δ ppm, *J* Hz): 0.87 (t, *J* = 6.9, 3H, CH_3_), 1.25–1.43 (m, 12H, 6 × CH_2_), 1.70 (m, 2H, βCH_2_), 2.77 (m, 2H, αCH_2_), 5.97 (s, 1H, C3-H), 7.36 (m, 1H, ArH), 7.72 (m, 1H, ArH), 7.86 (m, 1H, ArH), 8.12 (m, 1H, ArH), 11.43 (br s, 1H, OH); ^13^C-NMR (100 MHz, DMSO-d_6_, 70 °C, δ ppm): 14.22, 22.43, 27.77, 29.02, 29.08, 29.12, 29.25, 31.22, 31.66, 107.13, 115.45, 123.72, 125.30, 125.40, 132.12, 141.09, 153.83; HRMS *m*/*z* (ES+): calcd. for C_18_H_26_NO_2_^+^ [M+H]^+^ 288.1958, found 288.1954.

## Figures and Tables

**Table 1:** Isolated yield of products **4** and **5**, obtained according to Scheme 1.

Product	R^1^	R^2^	Conditions	Yield (%)
**4a**	C_6_H_5_	H	A	84
**4b**	*p*-MeOC_6_H_4_	H	A	80
**4c**	*p*-ClC_6_H_4_	H	A	72
**5a**	C_6_H_5_	OH	B	70
**5b**	*p*-MeOC_6_H_4_	OH	B	70
**5c**	*p*-ClC_6_H_4_	OH	C	82

**Table 2:** Antibacterial assay of the synthesized compounds at 100 µg/mL in DMSO, with 60 µL loading in 6 mm agar wells.

	Sterile Zone Diameter (mm) ^1^					
Product	*E. coli * ATCC 25922	*E. coli * ATCC 8739	*S. aureus * ATCC 25923	*S. aureus * ATCC 6538	*Enterococcus faecalis * ATCC 29212	*B. subtilis* NBIMCC 1208
**4a**	-	-	19	-	-	-
**4b**	-	-	11	-	-	-
**4c**	-	-	9	-	-	9
**5a**	-	15	25	-	-	15
**5b**	-	-	20	-	15	15
**5c**	15	15	19	16	15	15
**7**	-	-	14	-	-	9
**8**	14	15	27	14	14	20

^1^ Including 6 mm well diameter.

## Data Availability

The original contributions presented in the study are included in the article and Supplementary Materials. The raw NMR data is available for download at https://zenodo.org/doi/10.5281/zenodo.12749916. Further inquiries can be directed to the corresponding author/s.

## Supplementary Materials

The following supporting information can be downloaded at: https://www.mdpi.com/article/10.3390/molecules29153676/s1, File S1: NMR and ESI+ mass spectra of all final products.
