# Supplementary material for: Concise Synthesis of Pseudane IX, Its N-Oxide, and Novel Carboxamide Analogs with Antibacterial Activity
Source: Molecules. 2024 Aug 2;29(15):3676. doi: 10.3390/molecules29153676 (PMC11314064; doi:10.3390/molecules29153676)

Concise Synthesis of Pseudane IX, its *N*-Oxide and Novel Carboxamide Analogs with Antibacterial Activity

Plamen Angelov, Yordanka Mollova-Sapundzhieva, Francisco Alonso, Bogdan Goranov, Paraskev Nedialkov and Denitsa Bachvarova

Supplementary Information (NMR and Mass spectra of all final products)

Primary unprocessed data is available for download at https://doi.org/10.5281/zenodo.12749917

1. ^{1}H and ^{13}C NMR spectra:

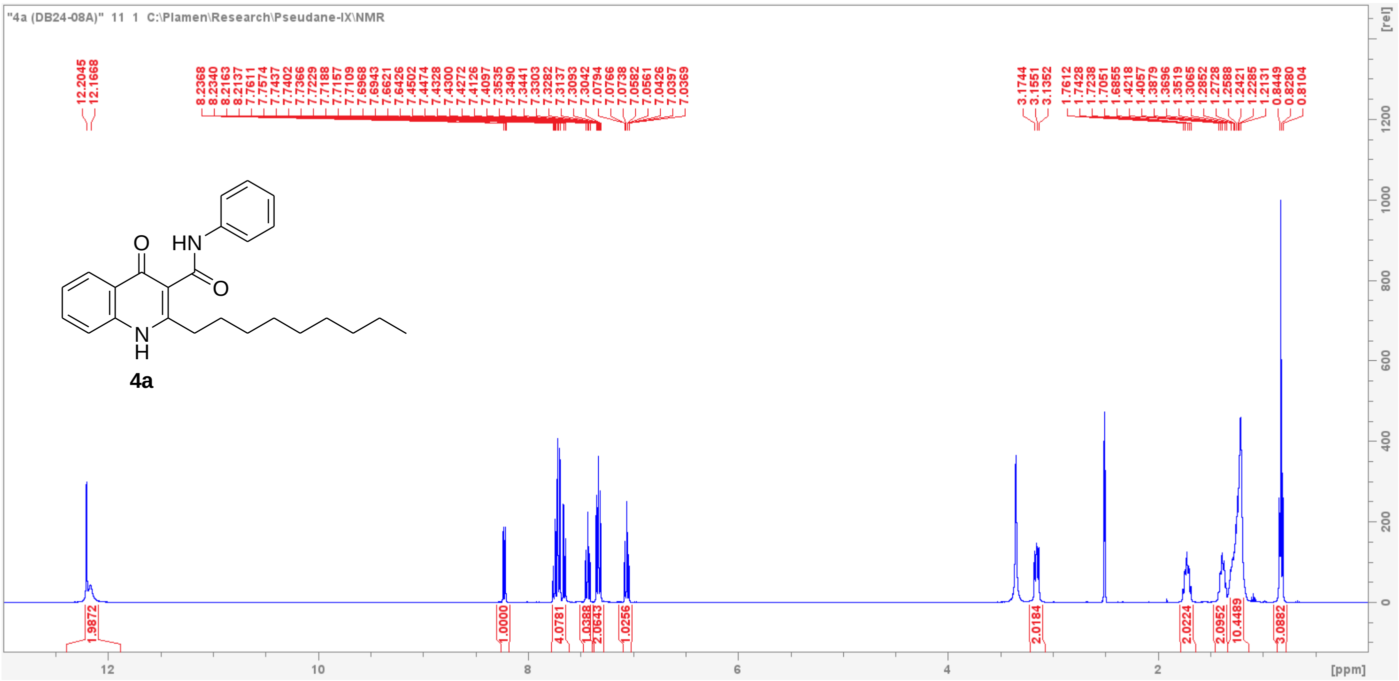

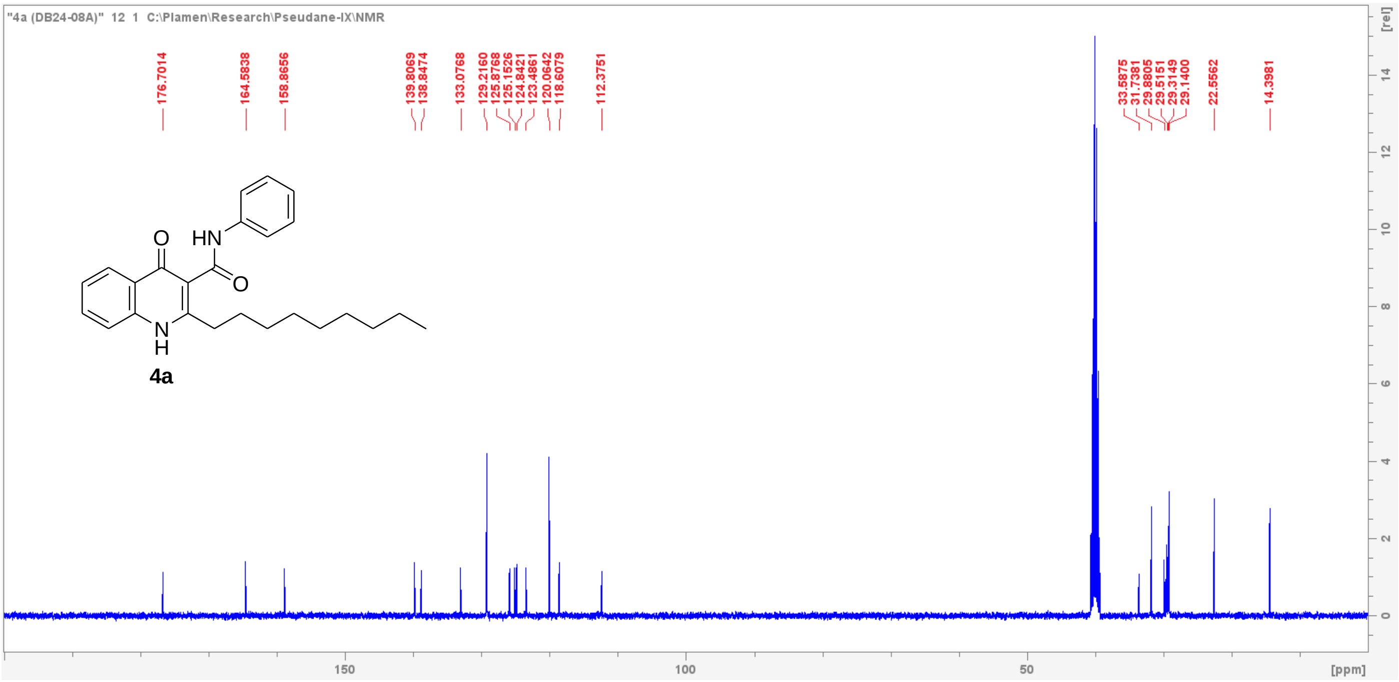

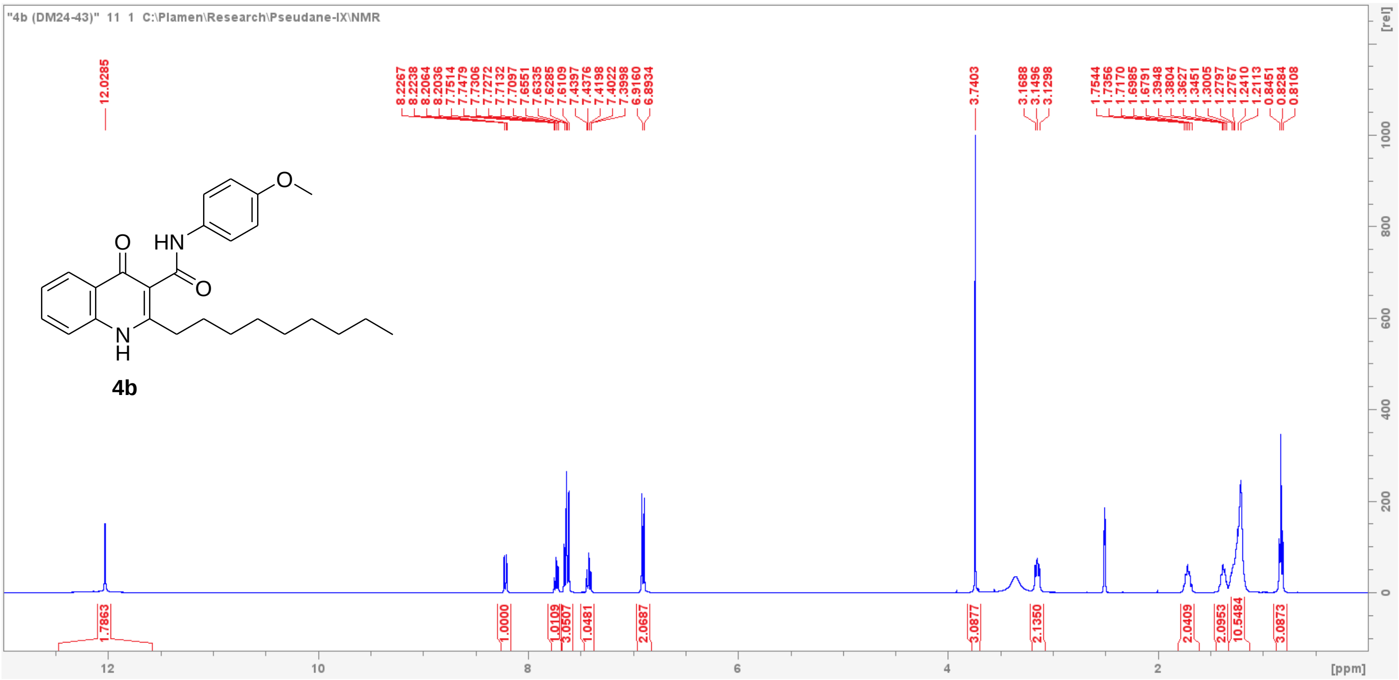
"4b (DM24-43)" 11 1 C:\Plamen\Research\Pseudane-IX\NMR
4b
[ppm]
[rel]

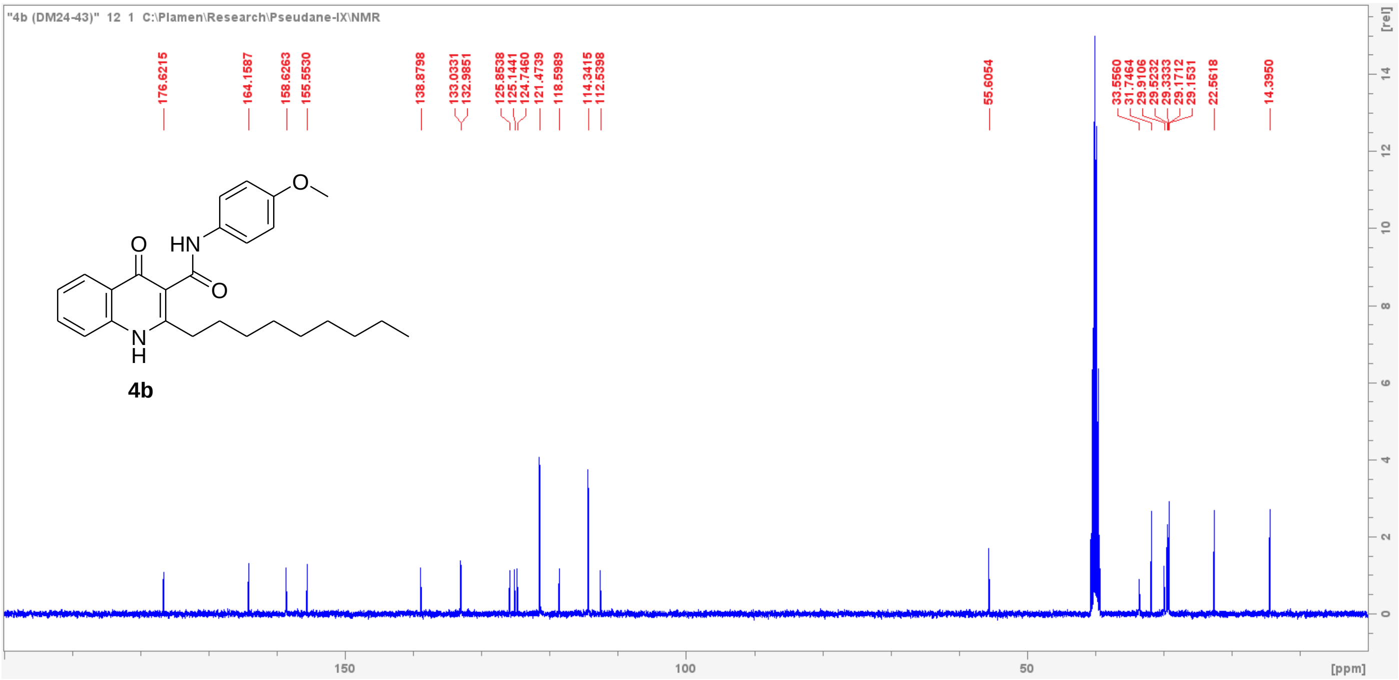
"4b (DM24-43)" 12 1 C:\Plamen\Research\Pseudane-IX\NMR
4b
[ppm]
[rel]

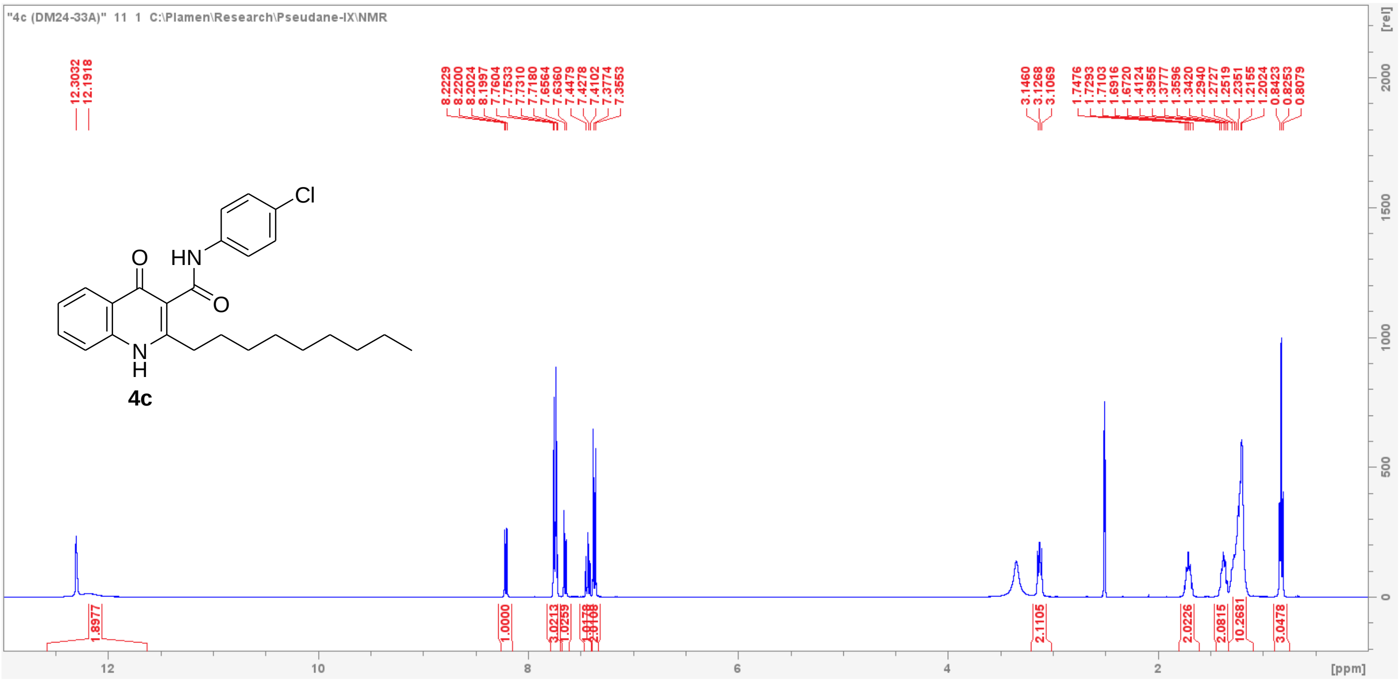
"4c (DM24-33A)" 11 1 C:\Plamen\Research\Pseudane-IX\NMR
12.3032
12.1918
8.2229
8.2200
8.2024
8.1997
7.7604
7.7533
7.7310
7.7180
7.6564
7.6360
7.4479
7.4278
7.4102
7.3774
7.3553
3.1460
3.1268
3.1069
1.7476
1.7293
1.7103
1.6916
1.6720
1.4124
1.3955
1.3777
1.3596
1.3420
1.2940
1.2727
1.2519
1.2351
1.2155
1.2024
0.8423
0.8253
0.8079
Cl
O
HN
O
N
H
4c
1.8977
1.0000
3.0213
1.0259
1.0178
2.0108
2.1105
2.0226
2.0815
10.2681
3.0478
[ppm]
[rel]

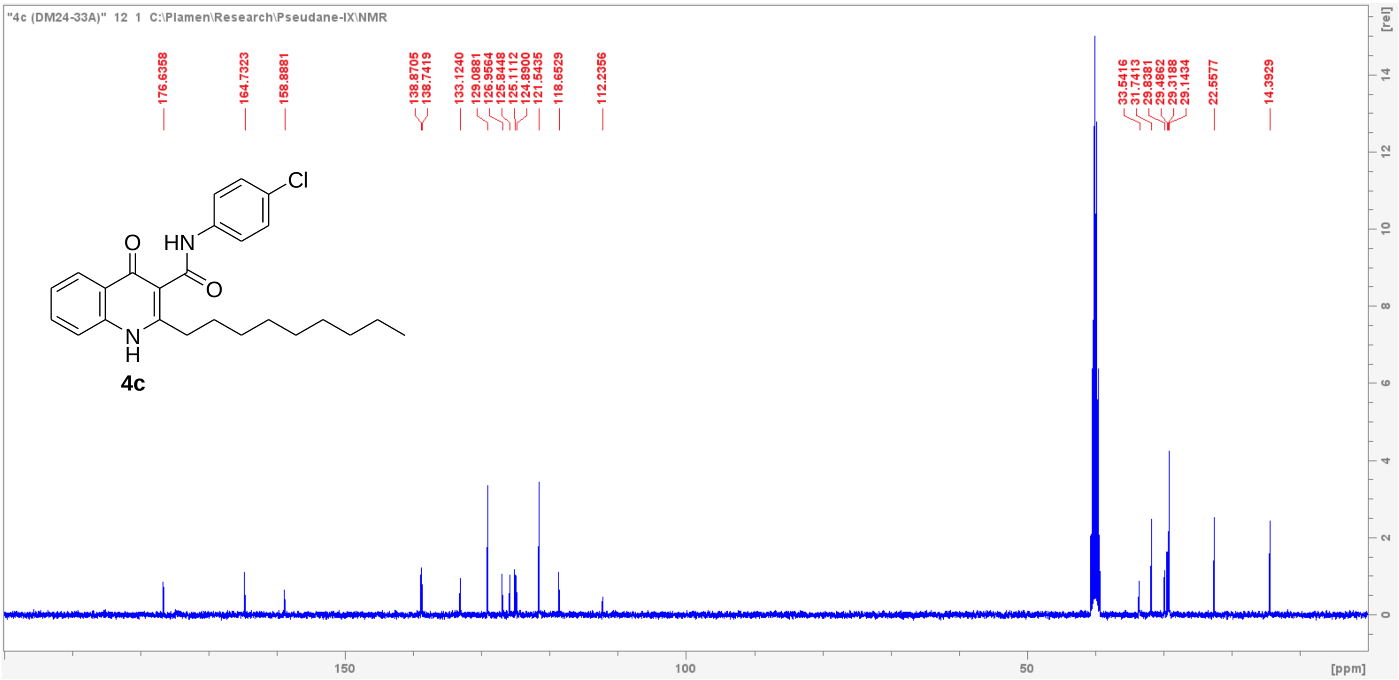
"4c (DM24-33A)" 12 1 C:\Plamen\Research\Pseudane-IX\NMR
176.6358
164.7323
158.8881
138.8705
138.7419
133.1240
129.0881
126.9564
125.8448
125.1112
124.8900
121.5435
118.6529
112.2356
33.5416
31.7413
29.8381
29.4862
29.3188
29.1434
22.5577
14.3929
Cl
O
HN
O
N
H
4c
[ppm]
[rel]

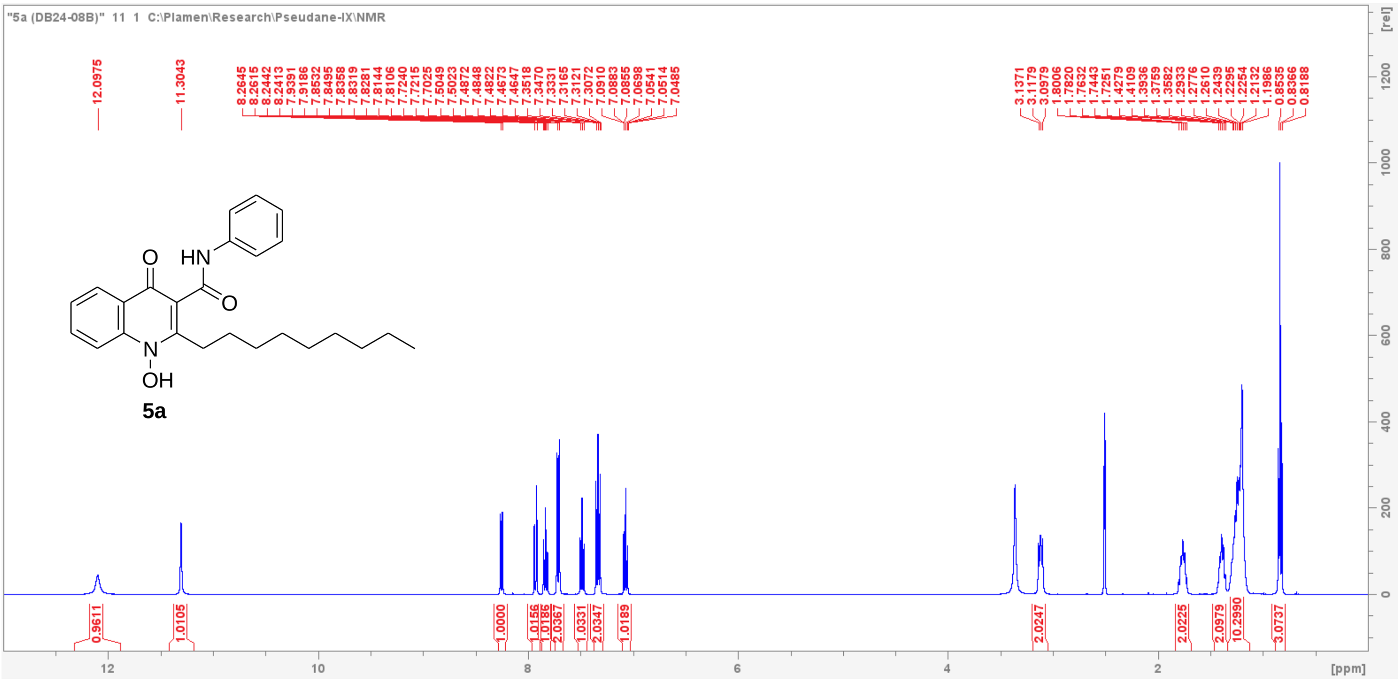
"5a (DB24-08B)" 11 1 C:\Plamen\Research\Pseudane-IX\NMR
12.0975
11.3043
8.2645 8.2615 8.2442 8.2413 7.9391 7.9186 7.8532 7.8495 7.8358 7.8319 7.8281 7.8144 7.8106 7.7240 7.7215 7.7025 7.5049 7.5023 7.4872 7.4848 7.4822 7.4673 7.4647 7.3518 7.3470 7.3331 7.3165 7.3121 7.3072 7.0910 7.0883 7.0855 7.0698 7.0541 7.0514 7.0485
3.1371 3.1179 3.0979 1.8006 1.7820 1.7632 1.7443 1.7251 1.4279 1.4109 1.3936 1.3759 1.3582 1.2933 1.2776 1.2610 1.2439 1.2295 1.2254 1.2132 1.1986 0.8535 0.8366 0.8188
O
HN
O
N
OH
5a
0.9611 1.0105 1.0000 1.0156 1.0186 2.0367 1.0331 2.0347 1.0189 2.0247 2.0225 2.0979 10.2990 3.0737
[ppm]
[rel]

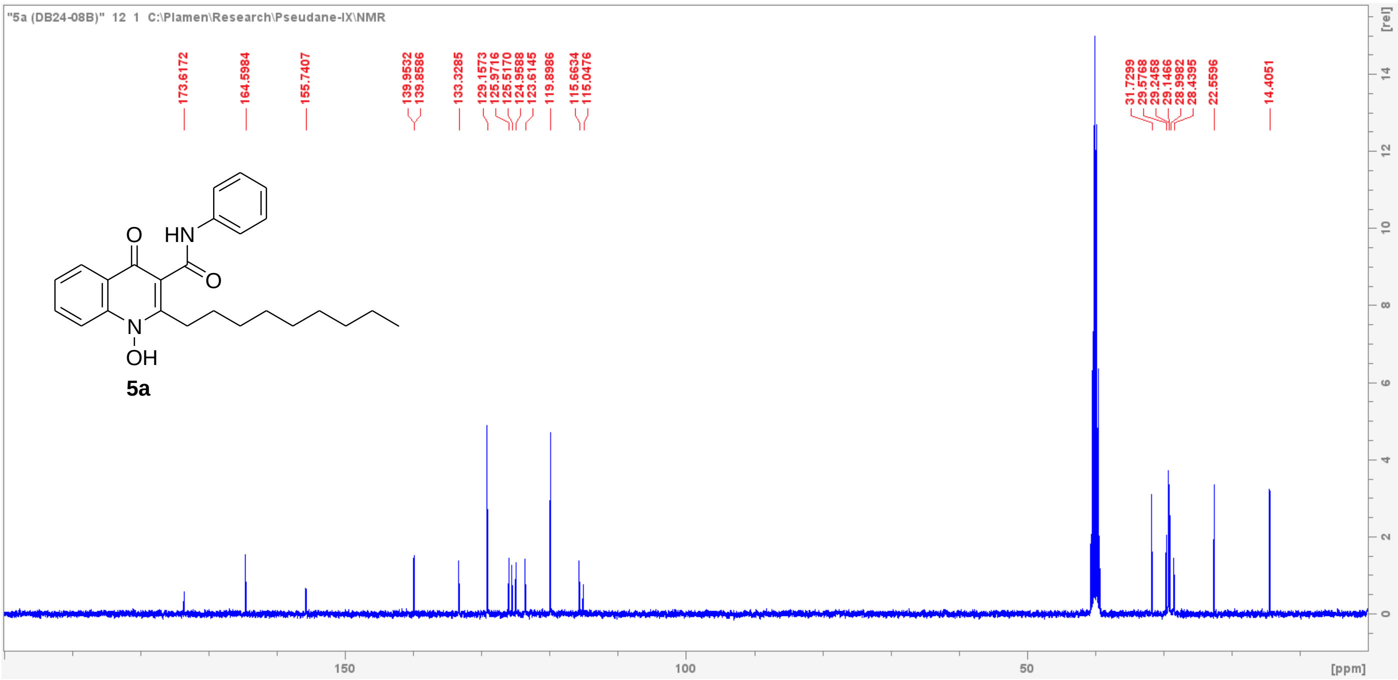
"5a (DB24-08B)" 12 1 C:\Plamen\Research\Pseudane-IX\NMR
173.6172
164.5984
155.7407
139.9532 139.8586
133.3285
129.1573 125.9716 125.5170 124.9588 123.6145
119.8986
115.6634 115.0476
31.7299 29.5768 29.2458 29.1466 28.9982 28.4355
22.5596
14.4051
O
HN
O
N
OH
5a
[ppm]
[rel]

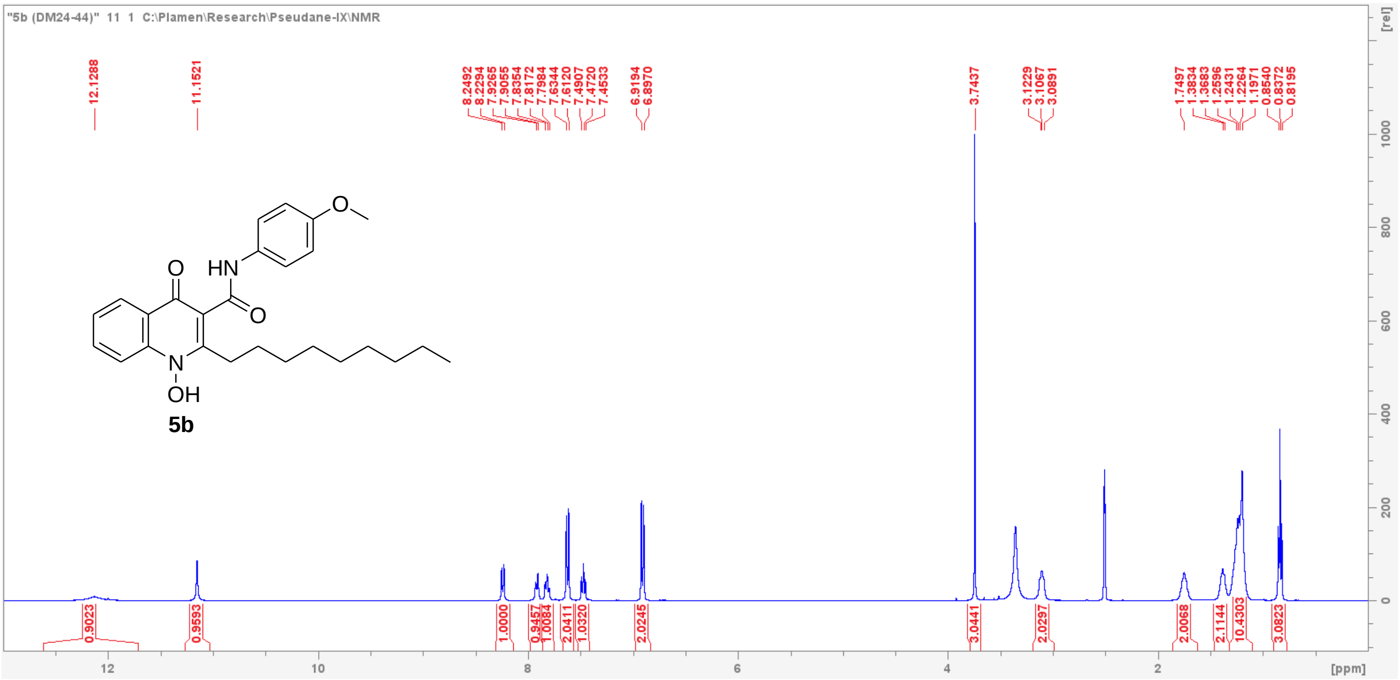
"5b (DM24-44)" 11 1 C:\Plamen\Research\Pseudane-IX\NMR
12.1288
11.1521
8.2492
8.2294
7.9265
7.9055
7.8354
7.8172
7.7984
7.6344
7.6120
7.4907
7.4720
7.4533
6.9194
6.8970
3.7437
3.1229
3.1067
3.0891
1.7497
1.3834
1.3683
1.2596
1.2431
1.2264
1.1971
0.8540
0.8372
0.8195
O
HN
O
O
N
OH
5b
0.9023
0.9593
1.0000
0.9457
1.0084
2.0411
1.0320
2.0245
3.0441
2.0297
2.0068
2.1144
10.4303
3.0823
12
10
8
6
4
2
[ppm]
[rel]

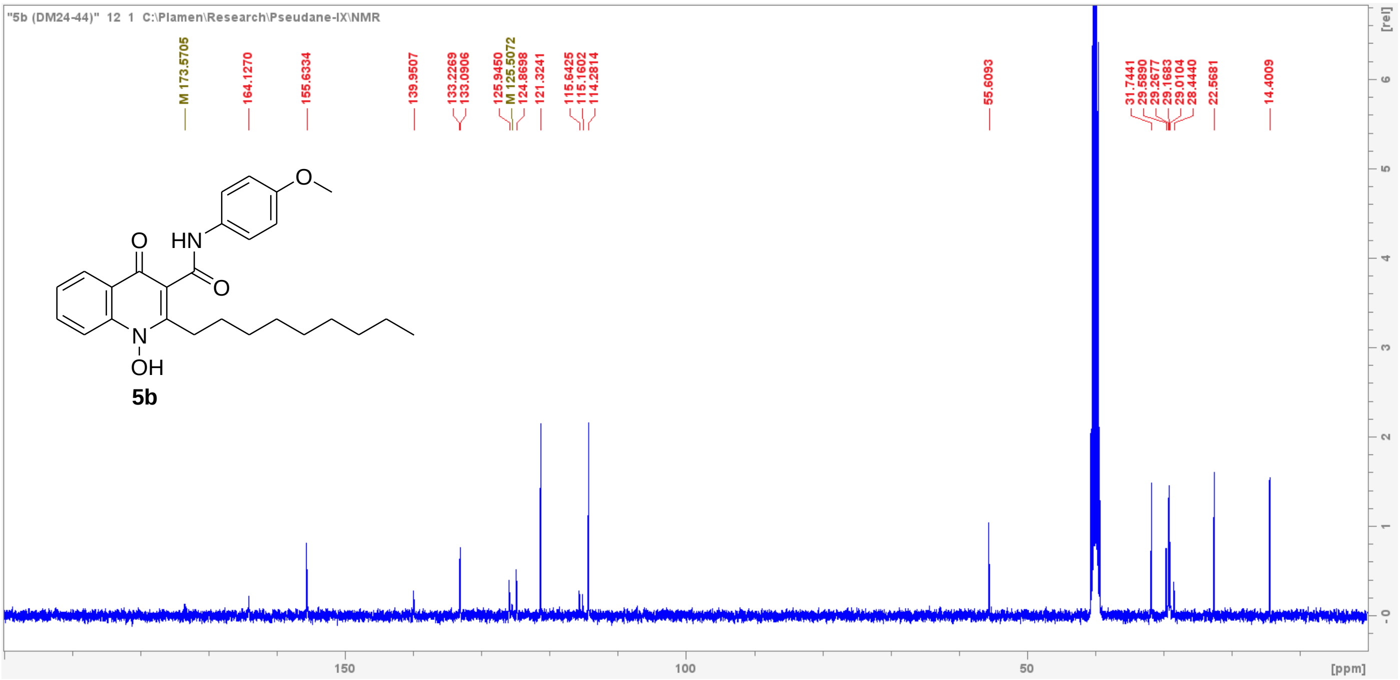
"5b (DM24-44)" 12 1 C:\Plamen\Research\Pseudane-IX\NMR
M 173.5705
164.1270
155.6334
139.9507
133.2269
133.0906
125.9450
M 125.5072
124.8698
121.3241
115.6425
115.1602
114.2814
55.6093
31.7441
29.5890
29.2677
29.1683
29.0104
28.4440
22.5681
14.4009
O
HN
O
O
N
OH
5b
150
100
50
[ppm]
[rel]

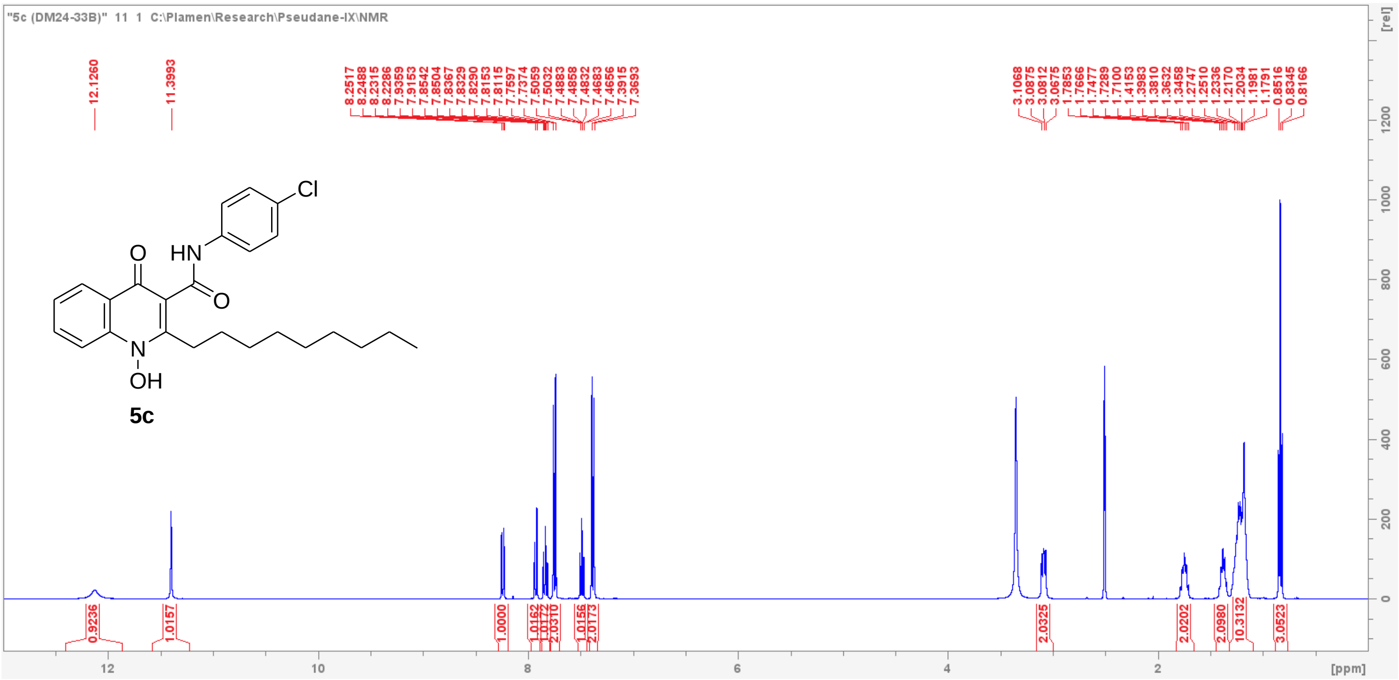
"5c (DM24-33B)" 11 1 C:\Plamen\Research\Pseudane-IX\NMR
12.1260
11.3993
8.2517
8.2488
8.2315
8.2286
7.9359
7.9153
7.8542
7.8504
7.8367
7.8329
7.8290
7.8153
7.8115
7.7597
7.7374
7.5059
7.5032
7.4883
7.4858
7.4832
7.4683
7.4656
7.3915
7.3693
3.1068
3.0875
3.0812
3.0675
1.7853
1.7666
1.7477
1.7289
1.7100
1.4153
1.3983
1.3810
1.3632
1.3458
1.2747
1.2510
1.2336
1.2170
1.2034
1.1981
1.1791
0.8516
0.8345
0.8166
Cl
HN
O
O
N
OH
5c
0.9236
1.0157
1.0000
1.0162
1.0172
2.0310
1.0156
2.0173
2.0325
2.0202
2.0980
10.3132
3.0523
[ppm]
[rel]

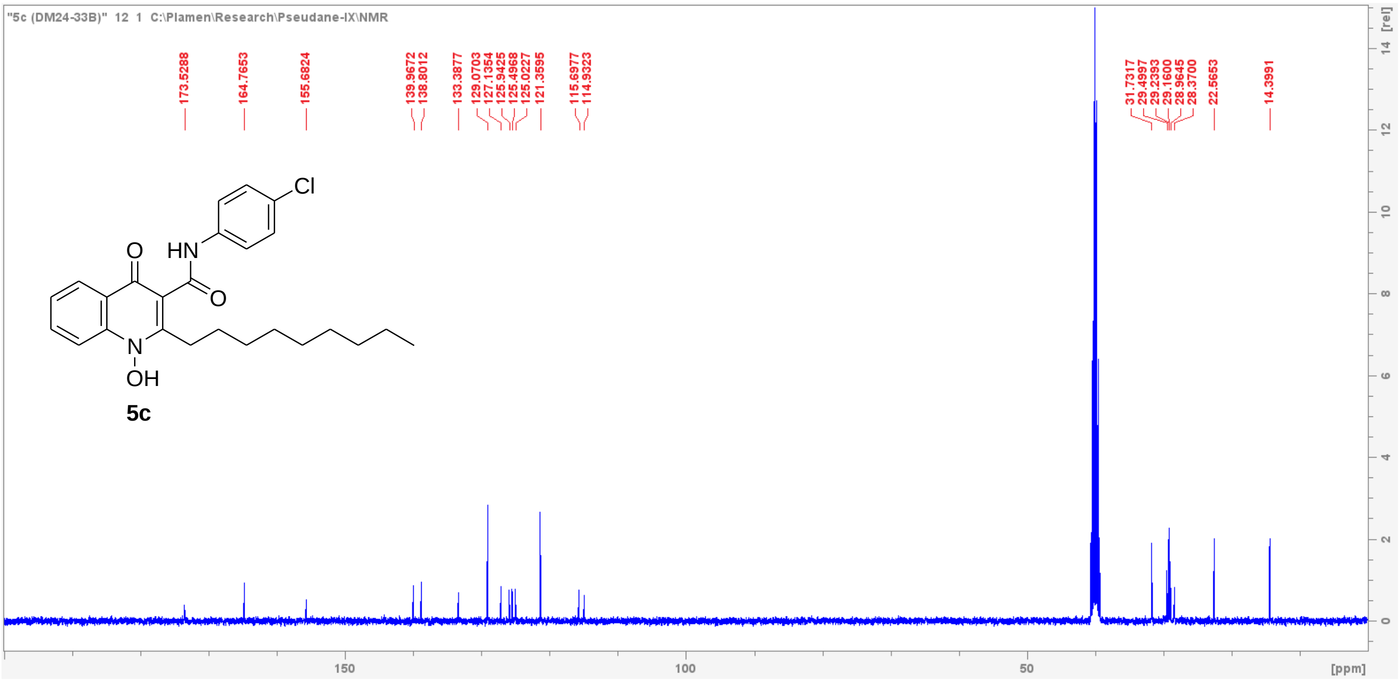
"5c (DM24-33B)" 12 1 C:\Plamen\Research\Pseudane-IX\NMR
173.5288
164.7653
155.6824
139.9672
138.8012
133.3877
129.0703
127.1354
125.9425
125.4968
125.0227
121.3595
115.6977
114.9323
31.7317
29.4997
29.2393
29.1600
28.9645
28.3700
22.5653
14.3991
Cl
HN
O
O
N
OH
5c
[ppm]
[rel]

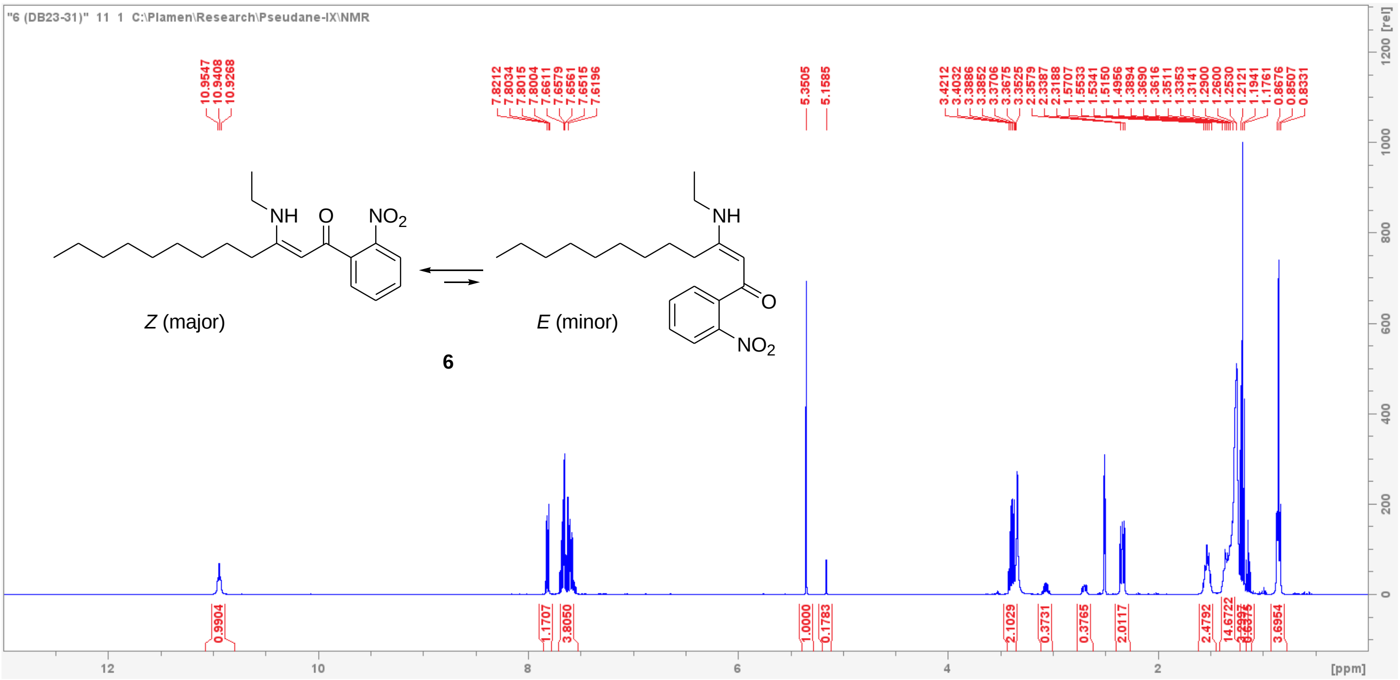
"6 (DB23-31)" 11 1 C:\Plamen\Research\Pseudane-IX\NMR
10.9547
10.9408
10.9268
7.8212
7.8034
7.8015
7.8004
7.6611
7.6579
7.6561
7.6515
7.6196
5.3505
5.1585
3.4212
3.4032
3.3886
3.3852
3.3706
3.3675
3.3525
2.3579
2.3387
2.3188
1.5707
1.5533
1.5341
1.5150
1.4956
1.3894
1.3690
1.3616
1.3511
1.3353
1.3141
1.2900
1.2600
1.2530
1.2121
1.1941
1.1761
0.8676
0.8507
0.8331
NH
O
NO_2
Z (major)
E (minor)
6
0.9904
1.1707
3.8050
1.0000
0.1783
2.1029
0.3731
0.3765
2.0117
2.4792
14.6722
3.6954
[ppm]
[rel]

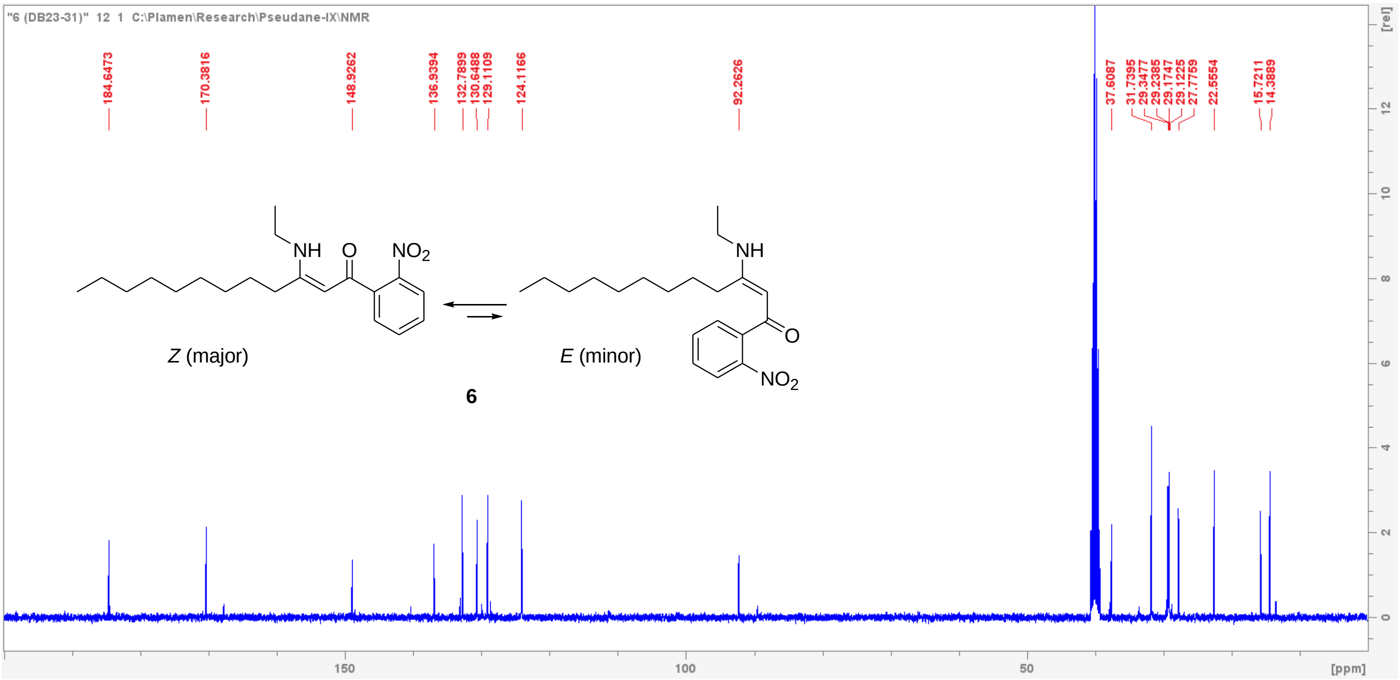
"6 (DB23-31)" 12 1 C:\Plamen\Research\Pseudane-IX\NMR
184.6473
170.3816
148.9262
136.9394
132.7899
130.6488
129.1109
124.1166
92.2626
37.6087
31.7395
29.3477
29.2385
29.1747
29.1225
27.7759
22.5554
15.7211
14.3889
NH
O
NO_2
Z (major)
E (minor)
6
150
100
50
[ppm]
[rel]

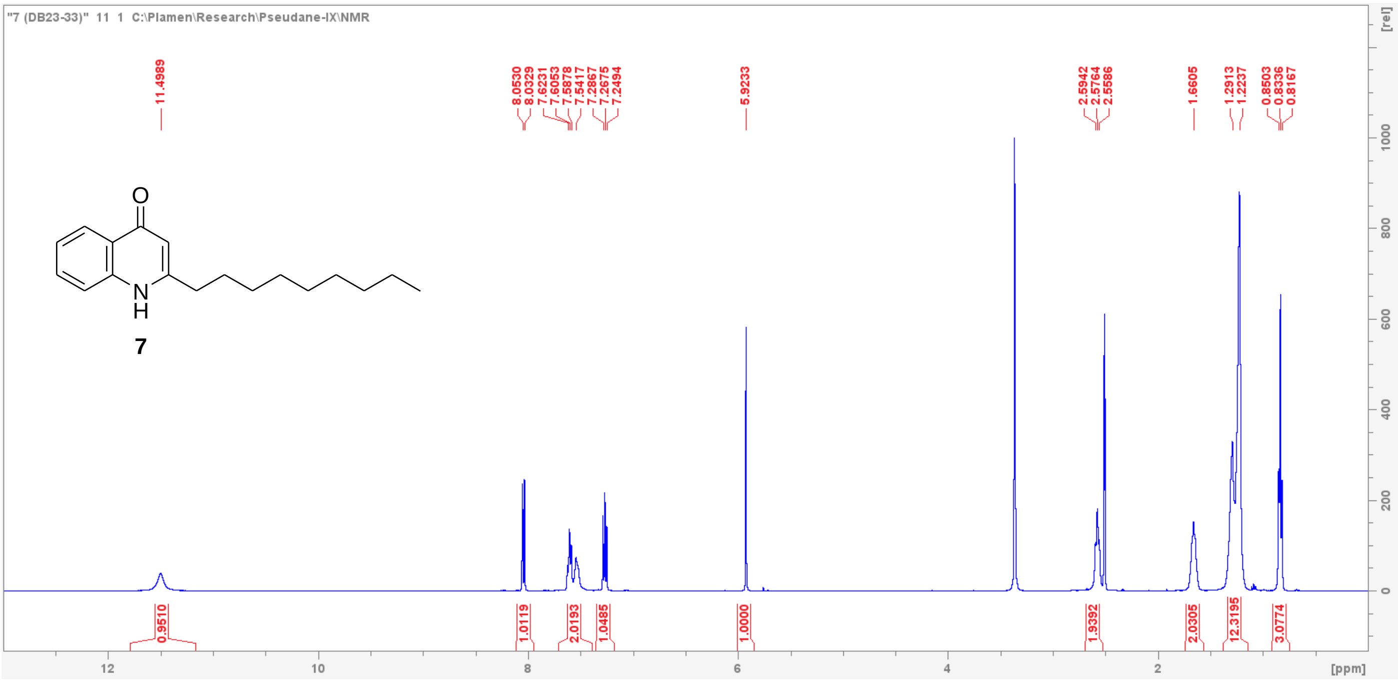
"7 (DB23-33)" 11 1 C:\Plamen\Research\Pseudane-IX\NMR
11.4989
8.0530
8.0329
7.6231
7.6053
7.5878
7.5417
7.2867
7.2675
7.2494
5.9233
2.5942
2.5764
2.5586
1.6605
1.2913
1.2237
0.8503
0.8336
0.8167
0.9510
1.0119
2.0193
1.0485
1.0000
1.9392
2.0305
12.3195
3.0774
O
N
H
7
[ppm]
[rel]

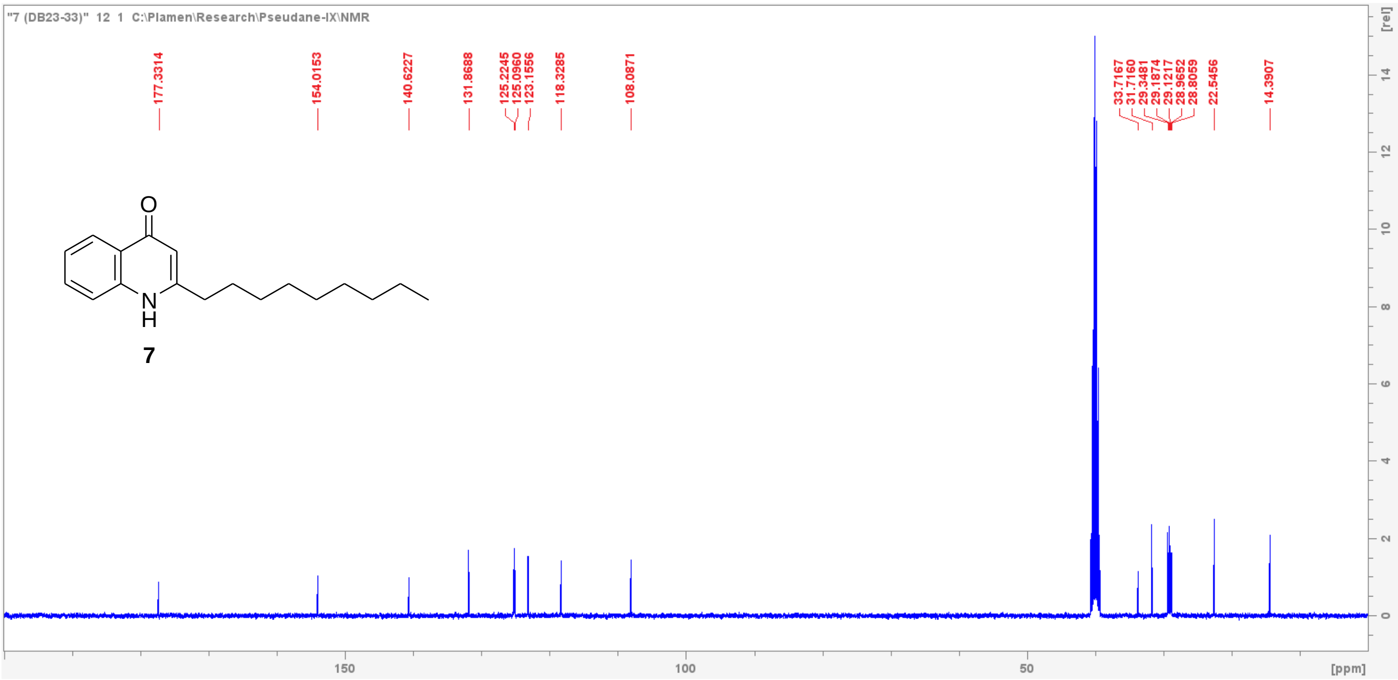
"7 (DB23-33)" 12 1 C:\Plamen\Research\Pseudane-IX\NMR
177.3314
154.0153
140.6227
131.8688
125.2245
125.0960
123.1556
118.3285
108.0871
33.7167
31.7160
29.3481
29.1874
29.1217
28.9652
28.8059
22.5456
14.3907
O
N
H
7
[ppm]
[rel]

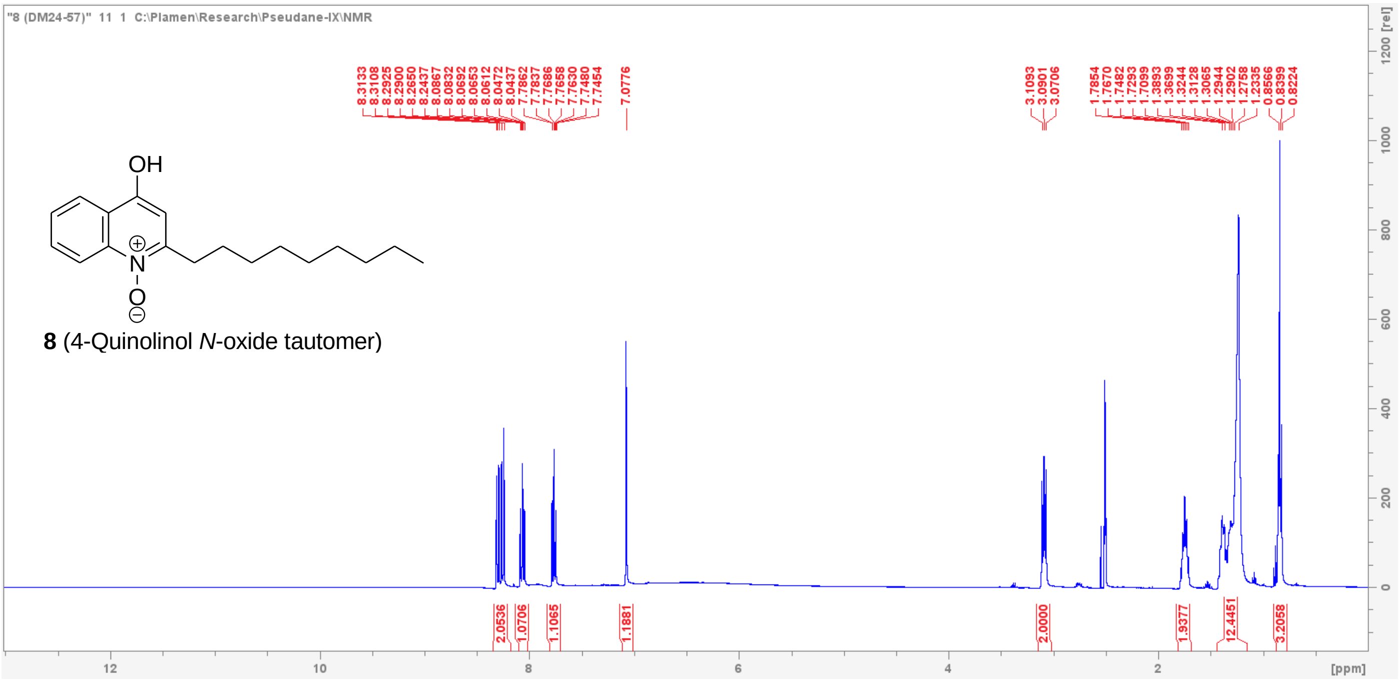
"8 (DM24-57)" 11 1 C:\Plamen\Research\Pseudane-IX\NMR
8.3133
8.3108
8.2925
8.2900
8.2650
8.2437
8.0867
8.0832
8.0692
8.0653
8.0612
8.0472
8.0437
7.7862
7.7837
7.7686
7.7658
7.7630
7.7480
7.7454
7.0776
3.1093
3.0901
3.0706
1.7854
1.7670
1.7482
1.7293
1.7099
1.3893
1.3699
1.3244
1.3128
1.3065
1.2944
1.2902
1.2758
1.2335
0.8566
0.8399
0.8224
OH
N
O
8 (4-Quinolinol N-oxide tautomer)
2.0536
1.0706
1.1065
1.1881
2.0000
1.9377
12.4451
3.2058
[ppm]
[rel]

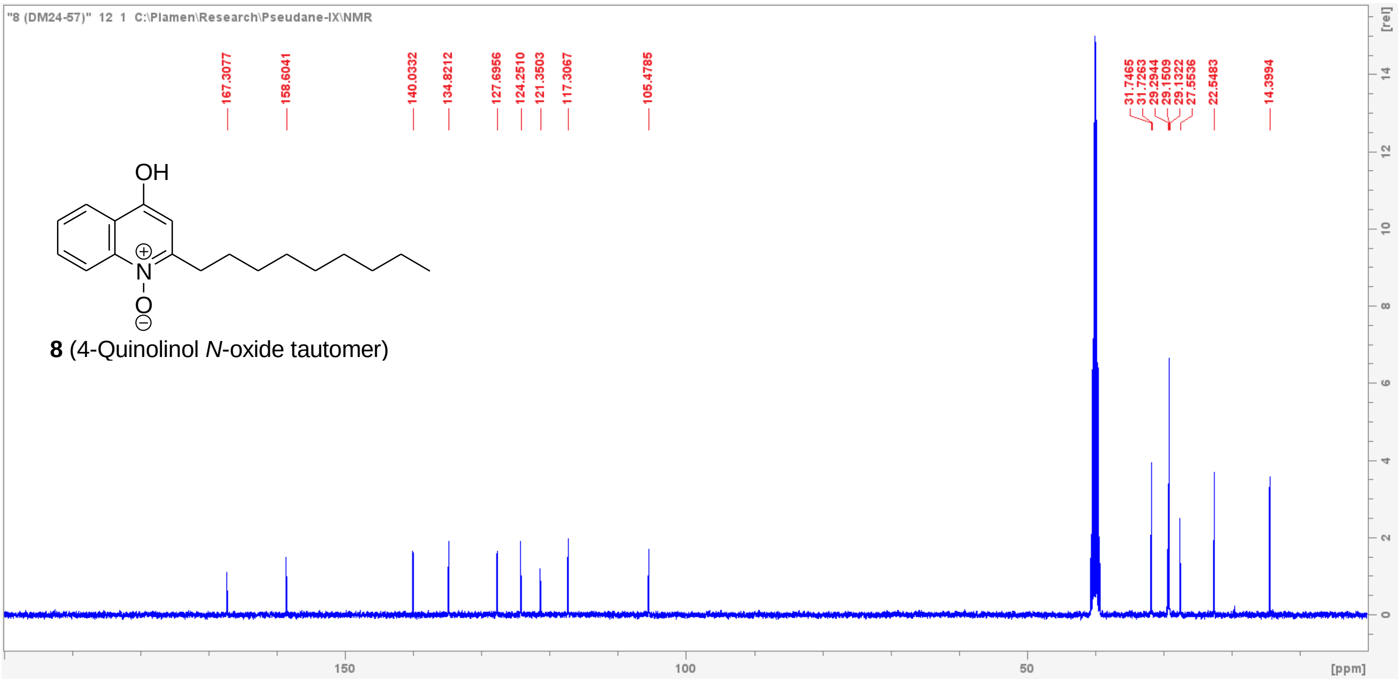
"8 (DM24-57)" 12 1 C:\Plamen\Research\Pseudane-IX\NMR
167.3077
158.6041
140.0332
134.8212
127.6956
124.2510
121.3503
117.3067
105.4785
31.7465
31.7263
29.2944
29.1509
29.1322
27.5536
22.5483
14.3994
OH
N
O
8 (4-Quinolinol N-oxide tautomer)
[ppm]
[rel]

(DMSO-d_6, 70 °C)

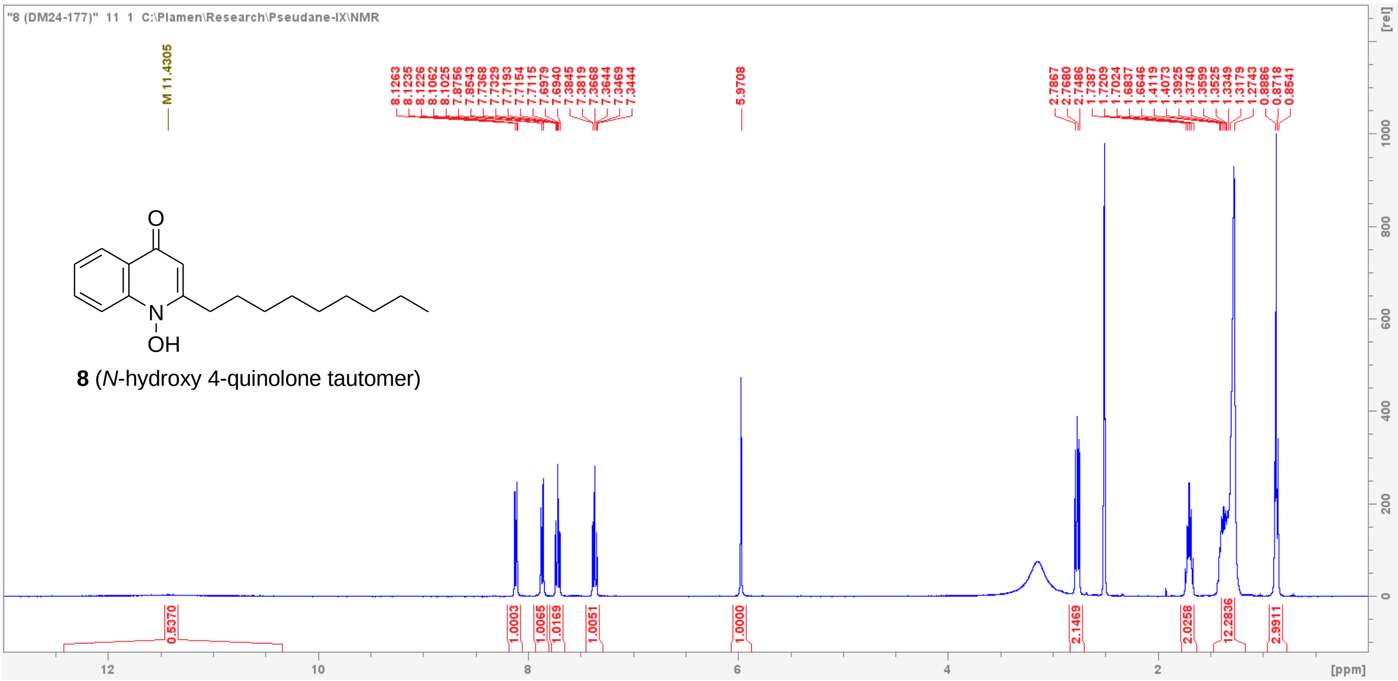
"8 (DM24-177)" 11 1 C:\Plamen\Research\Pseudane-IX\NMR
M 11.4305
8.1263 8.1235 8.1226 8.1062 8.1025 7.8756 7.8543 7.7368 7.7329 7.7193 7.7154 7.7115 7.6979 7.6940 7.3845 7.3819 7.3668 7.3644 7.3469 7.3444
5.9708
2.7867 2.7680 2.7486 1.7387 1.7209 1.7024 1.6837 1.6646 1.4119 1.4073 1.3925 1.3740 1.3599 1.3525 1.3349 1.3179 1.2743 0.8886 0.8718 0.8541
0.5370 1.0003 1.0065 1.0169 1.0051 1.0000 2.1469 2.0258 12.2836 2.9911
O
N
OH
8 (N-hydroxy 4-quinolone tautomer)
[rel]
[ppm]

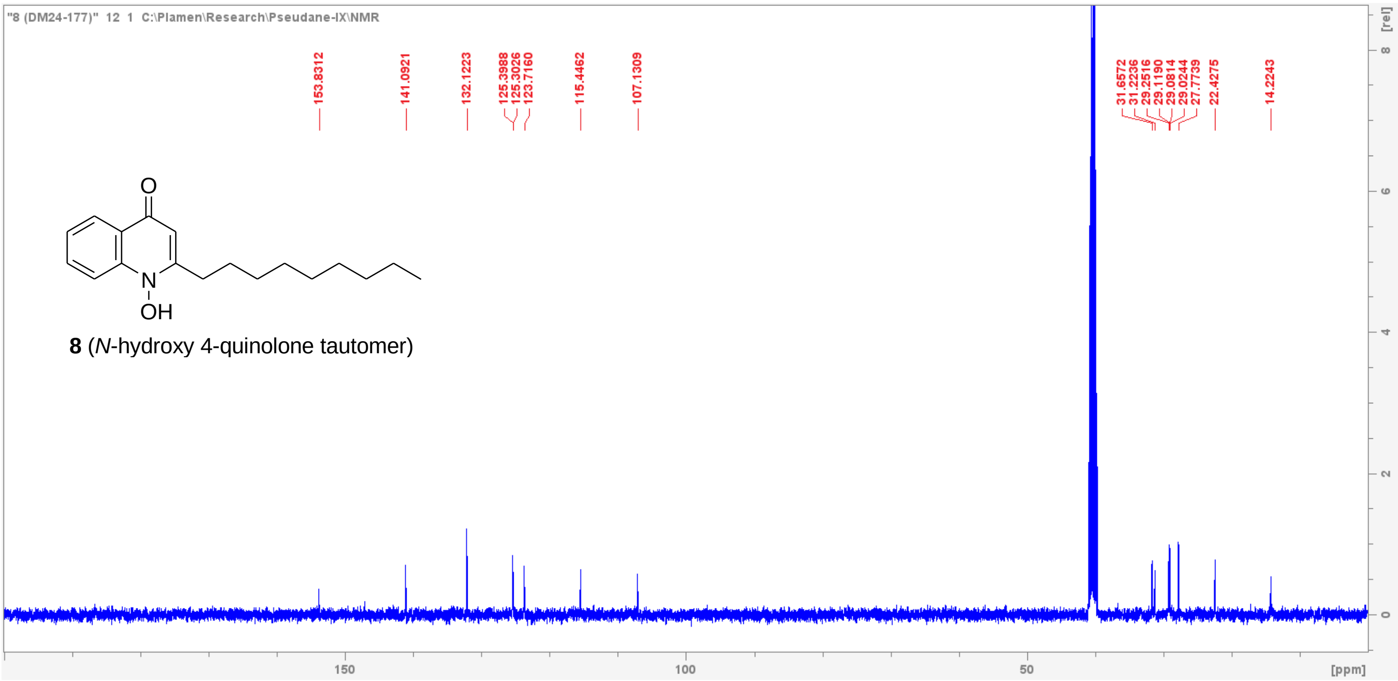
"8 (DM24-177)" 12 1 C:\Plamen\Research\Pseudane-IX\NMR
153.8312
141.0921
132.1223
125.3988 125.3026 123.7160
115.4462
107.1309
31.6572 31.2236 29.2516 29.1190 29.0814 29.0244 27.7739 22.4275
14.2243
O
N
OH
8 (N-hydroxy 4-quinolone tautomer)
[rel]
[ppm]

2. ESI(+) MS Data:

*2-Nonyl-4-oxo-1,4-dihydroquinoline-3-carboxylic acid phenylamide (**4a**):* calcd. for $C_{25}H_{31}N_2O_2^+$ $[M+H]^+$ 391.2380, found 391.2387

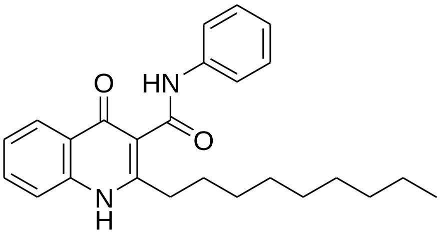

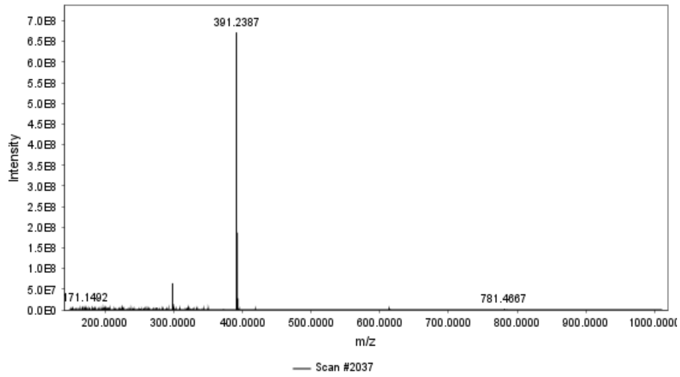

*2-Nonyl-4-oxo-1,4-dihydroquinoline-3-carboxylic acid 4-methoxyphenylamide (**4b**):* calcd. for $C_{26}H_{33}N_2O_3^+$ $[M+H]^+$ 421.2486, found 421.2481

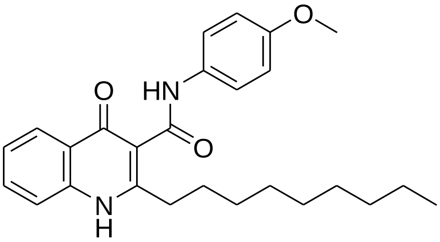

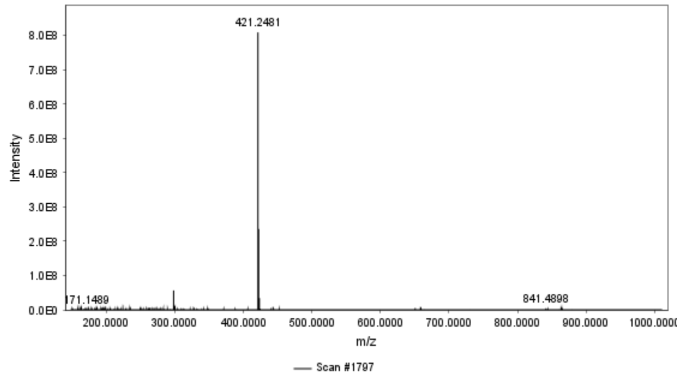

*2-Nonyl-4-oxo-1,4-dihydroquinoline-3-carboxylic acid 4-chlorophenylamide (**4c**):* calcd. for $C_{25}H_{30}ClN_2O_2^+$ $[M+H]^+$ 425.1990, found 425.1989

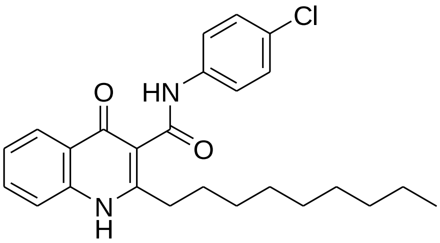

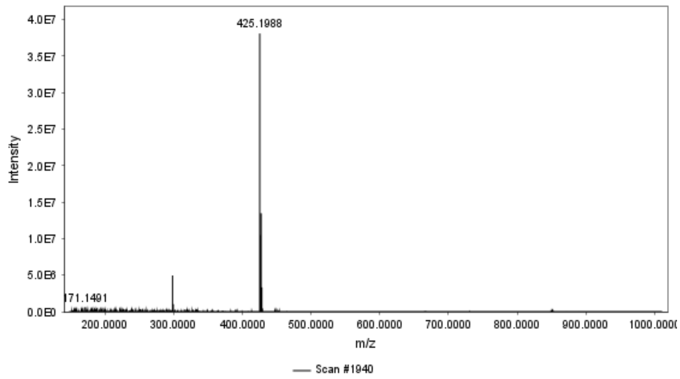

*1-Hydroxy-2-nonyl-4-oxo-1,4-dihydroquinoline-3-carboxylic acid phenylamide (**5a**):* calcd. for $C_{25}H_{31}N_2O_3^+$ $[M+H]^+$ 407.2329, found 407.2324

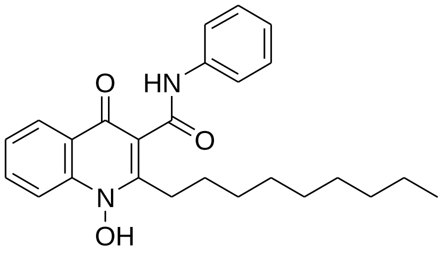

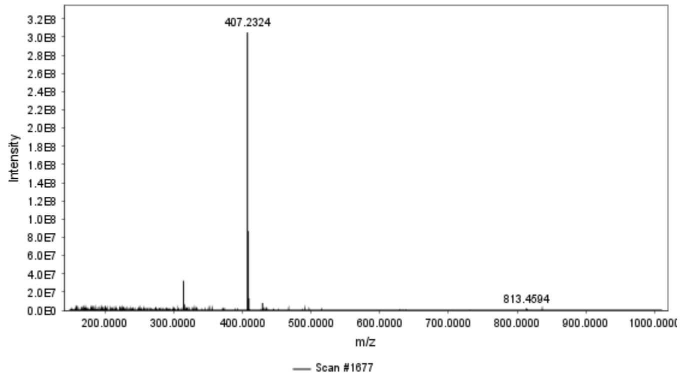

*1-Hydroxy-2-nonyl-4-oxo-1,4-dihydroquinoline-3-carboxylic acid 4-methoxyphenylamide (**5b**):* calcd. for $C_{26}H_{33}N_2O_4^+$ $[M+H]^+$ 437.2435, found 437.2434

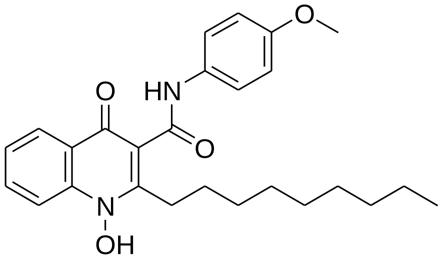

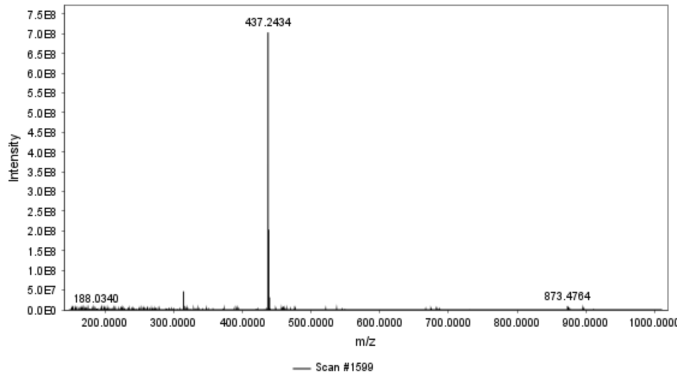

*1-Hydroxy-2-nonyl-4-oxo-1,4-dihydroquinoline-3-carboxylic acid 4-chlorophenylamide (**5c**):* calcd. for $C_{25}H_{30}ClN_2O_3^+$ $[M+H]^+$ 441.1939, found 441.1932

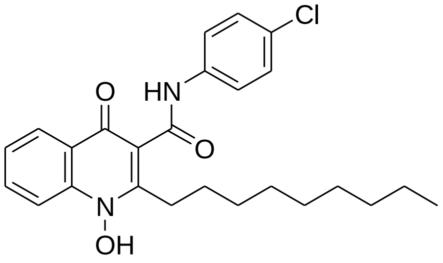

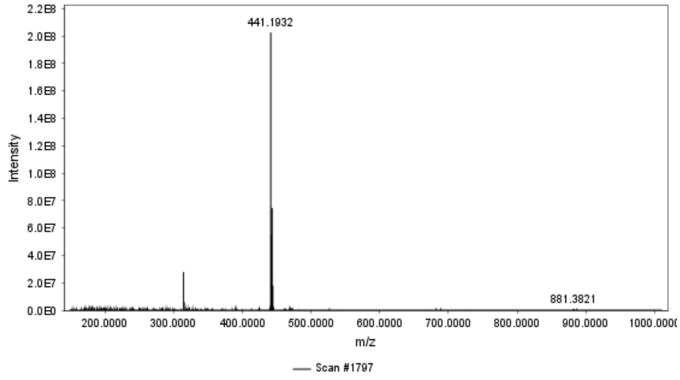

*2-Nonyl-1H-quinolin-4-one (Pseudane IX, **7**):* calcd. for $C_{18}H_{26}NO^+$ $[M+H]^+$ 272.2009, found 272.2007

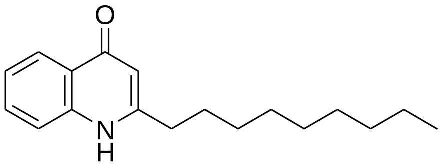

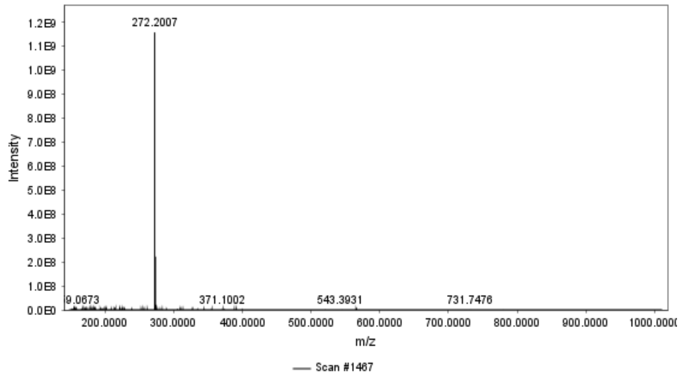

*1-Hydroxy-2-nonyl-(1H)-quinolin-4-one (**8**):* calcd. for $C_{18}H_{26}NO_2^+$ $[M+H]^+$ 288.1958, found 288.1954

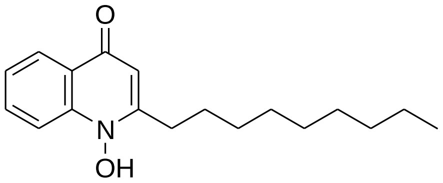

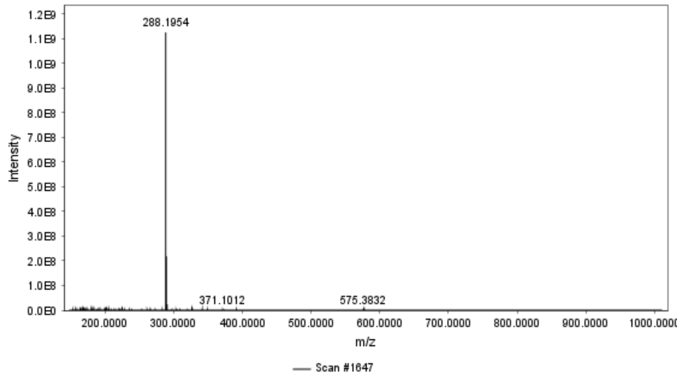

Supplement: Supplementary file 1 [file molecules-29-03676-s001.zip › molecules-3134925-supplementary.pdf]
